# Significant Effects of Oral Phenylbutyrate and Vitamin D3 Adjunctive Therapy in Pulmonary Tuberculosis: A Randomized Controlled Trial

**DOI:** 10.1371/journal.pone.0138340

**Published:** 2015-09-22

**Authors:** Akhirunnesa Mily, Rokeya Sultana Rekha, S. M. Mostafa Kamal, Abu Saleh Mohammad Arifuzzaman, Zeaur Rahim, Lamia Khan, Md. Ahsanul Haq, Khaliqu Zaman, Peter Bergman, Susanna Brighenti, Gudmundur H. Gudmundsson, Birgitta Agerberth, Rubhana Raqib

**Affiliations:** 1 International Centre for Diarrheal Disease Research, Bangladesh (icddr,b), Mohakhali, Dhaka-1212 Bangladesh; 2 Department of Laboratory Medicine, Clinical Microbiology, Karolinska Institutet, Karolinska University Hospital, Stockholm, Sweden; 3 National Institute of the Diseases of the Chest and Hospital, Mohakhali, Dhaka, Bangladesh; 4 Center for Infectious Medicine, Karolinska Institutet, Karolinska University Hospital, Stockholm, Sweden; 5 Biomedical Center, University of Iceland, 101 Reykjavik, Iceland; The Foundation for Medical Research, INDIA

## Abstract

**Background:**

Development of new tuberculosis (TB) drugs and alternative treatment strategies are urgently required to control the global spread of TB. Previous results have shown that vitamin D_3_ (vitD_3_) and 4-phenyl butyrate (PBA) are potent inducers of the host defense peptide LL-37 that possess anti-mycobacterial effects.

**Objective:**

To examine if oral adjunctive therapy with 5,000IU vitD_3_ or 2x500 mg PBA or PBA+vitD_3_ to standard chemotherapy would lead to enhanced recovery in sputum smear-positive pulmonary TB patients.

**Methods:**

Adult TB patients (n = 288) were enrolled in a randomized, double-blind, placebo-controlled trial conducted in Bangladesh. Primary endpoints included proportions of patients with a negative sputum culture at week 4 and reduction in clinical symptoms at week 8. Clinical assessments and sputum smear microscopy were performed weekly up to week 4, fortnightly up to week 12 and at week 24; TB culture was performed at week 0, 4 and 8; concentrations of LL-37 in cells, 25-hydroxyvitamin D_3_ (25(OH)D_3_) in plasma and *ex vivo* bactericidal function of monocyte-derived macrophages (MDM) were determined at week 0, 4, 8, 12 and additionally at week 24 for plasma 25(OH)D_3_.

**Results:**

At week 4, 71% (46/65) of the patients in the PBA+vitD_3_-group (*p* = 0.001) and 61.3% (38/62) in the vitD_3_-group (*p* = 0.032) were culture negative compared to 42.2% (27/64) in the placebo-group. The odds of sputum culture being negative at week 4 was 3.42 times higher in the PBA+vitD_3_-group (p = 0.001) and 2.2 times higher in vitD_3_-group (p = 0.032) compared to placebo. The concentration of LL-37 in MDM was significantly higher in the PBA-group compared to placebo at week 12 (*p* = 0.034). Decline in intracellular *Mtb* growth in MDM was earlier in the PBA-group compared to placebo (log rank 11.38, *p* = 0.01).

**Conclusion:**

Adjunct therapy with PBA+vitD_3_ or vitD_3_ or PBA to standard short-course therapy demonstrated beneficial effects towards clinical recovery and holds potential for host-directed-therapy in the treatment of TB.

**Trial Registration:**

clinicaltrials.gov NCT01580007

## Introduction

Tuberculosis (TB) is a global pandemic disease caused by *Mycobacterium tuberculosis* (*Mtb*) that is responsible for almost 9 million active pulmonary TB cases worldwide and about 1.5 million died from the disease in 2013 [1]. The prevalence of multidrug-resistant TB (MDR-TB) and extensively drug-resistant TB is high and converge with the pandemics of HIV and diabetes, which generates further problems with a lethal combination of diseases [2–4]. Despite this scenario, TB is a preventable and curable disease, although treatment of TB is currently long and requires multiple drugs that often have side effects which may range from mild to severe and may require tailored approach to individual cases. Development of new anti-TB drug has been very slow and most available drugs were developed more than 40 years ago [5]. Thus, there is a pressing need for development of new classes of novel drugs or repurposed drugs that can shorten the duration of treatment and combat infection with both susceptible and resistant strains of *Mtb*.

A series of *in vitro* studies have demonstrated that the active form of vitamin D, 1,25-dihydroxyvitamin D_3_ or 1,25(OH)_2_D_3_, induces the gene expression of beta-defensin 2 and human cathelicidin LL-37 [6, 7]. These peptides belong to two classes of antimicrobial peptides (AMP) produced in lung epithelial cells, monocytes/macrophages and neutrophils [8] that are able to suppress the growth of *Mtb* and modulate antimicrobial responses [9–11]. Active vitamin D, 1,25(OH)_2_D_3_ also induces autophagy in *Mtb*-infected macrophages/monocytes that can control the infection via an LL-37-dependent mechanism [12]. Accordingly, several studies have shown an association between vitamin D deficiency and an increased risk to develop active TB [13–16]. Altogether, these findings rekindled the interest of vitamin D as an adjunctive therapy to standard anti-TB treatment [17].

There is growing evidence that adjunct host-directed therapies, could serve as novel approaches to improve standard TB treatments [18–20]. We have earlier shown that treatment with sodium butyrate enhances cathelicidin expression in a colonic epithelia cell line [21] and *in vivo* in colonic epithelium in a rabbit model of shigellosis, which resulted in rapid clinical recovery and a concomitant decline in bacterial load in stool [22]. Similarly, we have further shown that sodium 4-phenylbutyrate (PBA), a registered drug used for the treatment of urea cycle disease [23] induces LL-37 expression in a bronchial epithelial cell line and *in vivo* in the rabbit shigellosis model [24, 25]. A synergistic effect in the induction of LL-37 has also been demonstrated between PBA and 1,25(OH)_2_D_3_ [24]. In a proof-of-concept study, we showed that supplementation with a combination of PBA and vitamin D to healthy adults enhanced LL-37 expression and intracellular killing of *Mtb* in macrophages *ex vivo* [26].

In this clinical trial, we aimed to test our hypothesis that oral adjunct therapy with PBA and/or vitD_3_ administrated to patients with active pulmonary TB would increase LL-37 expression in macrophages and other immune cells and eventually increase elimination of *Mtb* from the host. Thus, we conducted a randomized clinical trial to examine if PBA or vitD_3_ separately or in combination as adjunct treatment to standard chemotherapy could enhance clinical recovery in newly diagnosed sputum smear-positive pulmonary TB patients. Primary outcomes of the trial included the proportion of TB patients who became culture negative at week 4 and also assessment of clinical endpoints at week 8. Secondary outcome measures included time to sputum smear conversion, radiological changes, plasma 25(OH)D_3_ levels, expression of LL-37 in immune cells and killing of intracellular *Mtb* by infected MDM.

## Methods

### Clinical trial study participants

Patients with newly diagnosed sputum smear-positive pulmonary TB were recruited from the National Institute of the Diseases of the Chest and Hospital (NIDCH) in Dhaka, Bangladesh, after providing written informed consent. NIDCH is a government supported research institute and hospital where the majority of the patients are of low socioeconomic status. Inclusion criteria: both males and females (age ≥18 years) with a newly diagnosed sputum smear-positive TB who consented to study enrollment. Exclusion criteria: pregnancy and lactation, relapse TB, HIV infection, hypercalcaemia, regular intake of vitamin D, known concomitant chronic illness such as diabetes, cardiovascular, hepatic and renal diseases and malignancy. Patients with suspicion of prolonged drug abuse were also excluded. Information such as history of contact with active TB cases, duration of illness, BCG vaccination and tuberculin skin test status were recorded.

The study was approved by the Research and Ethical Review Committees at the International Centre for Diarrheal Disease Research, Bangladesh (icddr,b). The trial was registered with ClinicalTrials.gov (registration number NCT01580007) in April 2012 whereas patient recruitment started in December 2010. The reason for not registering the clinical trial before the recruitment of the first patient was that the authors were not aware of journal requirements for prospective registration. Importantly, the delay in trial registration did not have an impact on the study design or on the analysis or presentation of the results (see attached protocol).

### Study design and interventions

The study was a randomized, double blind, placebo controlled 4-arm intervention trial with adjunct therapy with PBA and/or vitD_3_ for 2 months. TB patients received directly observed treatment, short-course (DOTS) of a 4 fixed-dose-combination (4-FDC) drugs for 2 months followed by 2-FDC for the next 4 months. The 4-FDC consists of Rifampicin 150mg + Isoniazid 75mg + Pyrazinamide 400mg + Ethambutol 275mg/tablet while the 2-FDC consists of Rifampicin 150mg + Isoniazid 75mg/tablet. The company EM-Partners AB (Råå, Sweden) prepared tablets containing the adjunctive study drug (4-phenylbutyrate (PBA) (Tributyrate®) and identical tablets that contained the placebo (tablet bulking agents or excipient). Vitamin D_3_ (vit D_3_) (Vigantol oil) and placebo (Miglyol oil) were obtained from Merck KGaA in Darmstadt, Germany through Popular Pharmaceuticals Ltd in Bangladesh. After enrollment, patients were randomized to the following adjunct treatment arms in a 2x2 factorial design and received oral doses of either: (1) placebo PBA and placebo vitD_3_ or (2) 500 mg twice daily of PBA and placebo vitD_3_ or (3) placebo PBA and 5000 IU of vitD_3_ (Cholecalciferol) once daily or (4) PBA combined with vitD_3_ (PBA+vitD_3_).

The dose of PBA was chosen based on a recent study from our group where 500 mg PBA given twice daily to healthy volunteers proved to be the optimal oral dose to induce LL-37 and enhance mycobactericidal activity in monocyte-derived macrophages (MDM) obtained from treated individuals [26]. The dose of vitD_3_ was selected based on our previous findings that a weekly dose of 35,000 IU of vitD_3_ given for 2–3 months to pregnant women in the 3^rd^ trimester was found to be safe and also raised maternal 25(OH)D_3_ concentrations in serum significantly in most women without any adverse effects [27]. Moreover, there is evidence that daily doses up to 10,000 IU/day for several months have not lead to adverse effects of changes in serum calcium [28].

### Randomization and blinding

Independent assistants from the Hospital pharmacy of icddr,b prepared the study medication packs (PBA and placebo tablets; with identical appearance, color and taste), and labeled these tablets with a randomization number corresponding to the computer-generated randomization sequence. Similarly, Popular Pharmaceuticals Ltd. labeled bottles for vitD_3_ (Vigantol oil and Miglyol placebo oil; with identical appearance, color and taste) with the provided randomization number. To control and balance for the influence of gender a computer-generated stratified block randomization method was used to randomize participants into four groups that would result in equal sample sizes including 4x72 = 288 patients. The randomized block procedure was performed as follows: (1) a block size of four was chosen at two levels: male and female, (2) possible balanced combinations with four subjects were calculated as 24 blocks and (3) blocks were randomly chosen to determine the assignment of all 288 participants. This randomization protocol resulted in 72 participants (36 males and 36 females) in each of the four treatment groups. Treatment allocation was concealed from patients, study investigators and staff.

### Sample size

No published data is available on the culture conversion of drug-sensitive TB patients at week 4 or week 8 in Bangladesh. Data on smear conversion of Bangladeshi TB patients are available from a publication from 1998 [29] which shows 85% smear conversion at 1 month (4 weeks) following anti-TB treatment. After 2 months of anti-TB chemotherapy close to 99% patients became sputum smear negative. Thus, to observe treatment effect of adjunctive therapy on smear conversion in TB patients at 2 months is also not useful. Using these smear conversion data the sample size becomes impractically large (e.g. 726/arm). For these reasons, in absence of data for powering the trial according to the primary outcome findings from our preclinical studies [25] and TB scores reported by Wejse et al [30] were used for sample size calculation. Sample size calculations were made based on: i) preclinical outcome as a surrogate marker for sputum conversion: The mean difference in expression of the rabbit cathelicidin CAP-18 in the gut mucosal tissue of untreated *Shigella*-infected rabbits and infected rabbits compared to PBA-treated infected rabbits was 6.94 with an expected standard deviation of 3.5. Allowing for 4 groups and expecting a 30% increase in the expression of human cathelicidin LL-37 in tissues when treated with PBA+vitD_3_ along with standard anti-TB treatment (from 6.94 to 9.04), at a 5% level of significance and 80% power the sample size would be 62 per group. Considering a loss to follow-up of 15%, the sample size in each group would be 72 with a total of 288 patients. ii) A composite clinical score: In a superiority design, with a 50% reduction of clinical scores occurring at 2 month post anti-TB treatment [30], we assume that 25% reduction will take place after one month. A sample size of 212 patients (53 in each arm) would provide 80% power to demonstrate that each treatment will result in a 25% reduction of the clinical scores after 4 weeks of adjunct therapy (from 6.5 to 4.88) with expected standard deviation of 2.3 [30] making an allowance for 15% attrition rate at 5% level of significance. The highest sample size was considered. For sputum smear conversion, the study may be underpowered.

### Outcome measures

Primary outcomes of the trial included assessment of both microbiological and clinical endpoints. The microbiological outcome was measured as the proportion of TB patients who became culture negative at week 4, while effect size was assessed for major clinical endpoints (cough remission, reduction in lung involvement in chest x-ray, normalization of fever and weight gain) at week 8. Secondary outcome measures included time to sputum smear conversion, radiological findings, concentrations of 25(OH)D_3_ in plasma, immunological status measured as the expression of the antimicrobial peptide LL-37 in immune cells and also as killing of intracellular *Mtb* by MDM *ex vivo*.

### Patient safety monitoring, adverse events (AE) and serious adverse events (SAE)

A data and safety monitoring board (DSMB) was formed that conducted three meetings (before initiation of the study, interim and after completion) to review the results and to advice on the safety to continue or stop the trial after occurrence of a Serious adverse events (SAE) if any. Adverse events (AE) included hypercalcaemia (albumin adjusted plasma calcium >10.5 mg/dL), arthralgia, hepatitis/jaundice, vomiting, anemia, joint pains, body ache, abdominal pain, headache, malaise, itching, dyspepsia, vertigo, and other non-serious AEs, particularly during the first 8 weeks after start of adjunctive treatment. SAE comprised death, hospitalization or life-threatening conditions. The primary safety endpoint was hypercalcaemia (corrected calcium >10.5 mg/dL).

### Procedures

Clinical assessments and sputum microscopy examinations were performed weekly up to week 4, and consecutively at week 6, 8, 10, 12 and 24. Chest radiographs at NIDCH were examined at week 0, 8, 12 and 24. Sputum culture was performed at NIDCH at week 0, 4 and 8 while drug sensitivity testing was performed at icddr,b. Blood samples were collected at week 0, 4, 8, 12 and 24. Hemoglobin, erythrocyte sedimentation rate (ESR), total and differential counts were determined in the whole blood samples. Concentration of 25(OH)D_3_, calcium, albumin and C-reactive protein (CRP) were measured in plasma at all time points. MDM and non-adherent lymphocytes were separated from whole blood at week 0, 4, 8 and 12 and used for *in vitro* experiments.

### *Mtb* microscopy, culture and drug susceptibility testing

Acid-fast bacilli (AFB) in patient’s sputum samples were detected with Ziehl-Neelsen staining and direct smear microscopy. To assess *Mtb* growth in sputum cultures, sputum samples were inoculated onto slopes of Lowenstein-Jensen (LJ) media. Bacterial cultures were incubated at 37°C and monitored for 6 to 8 weeks or until colonies were detected. Drug susceptibility tests (DST) were performed in LJ media by the minimum inhibitory concentration method [31]. DST was performed for isoniazid, rifampicin, ethambutol and streptomycin.

### TB score

Clinical assessments were performed by the study doctor assisted by a nurse on scheduled visits and were used to calculate numerical clinical scores as previously described [30, 32]. The clinical score defined as a TB score is an assessment tool developed by clinicians/ investigators to measure changes in clinical symptoms of the TB patients in an unbiased and objective manner. The TB score (**S1 Table**) allocated points for self-reported symptoms (cough, shortness of breath/dyspnea, chest pain, haemoptysis, anorexia), and clinical signs (fever, anemia (<11 g/dl), tachycardia, auscultatory findings) as reported by study doctors. Weight gain was minimum (<8.5%) for the Bangladeshi TB patients and therefore not used in the scoring system. The TB score was determined at week 0–4, 6, 8, 10, 12 and 24.

In addition to the TB score, chest x-ray findings were assessed and scored as previously reported [32, 33]. Since chest x-ray was performed only at week 0, 8, 12 and 24 following the National TB program guidelines, a TB score including chest x-ray was determined separately for these four time points. For the chest x-ray analysis, each lung field was divided into three zones, upper, mid and lower zones. Presence of nodules, patchy or confluent consolidation and cavitation were recorded for each of the three zones. The size of nodules appearing as round or irregular were recorded in millimeters (small nodule, 1 to 2 mm; large nodules, >2 mm) and total cavity size was recorded in centimeters. The presence of effusion was also determined and the effusion volume was estimated by visual assessment as the percentage of a given lung field. Similarly, the extent of opacification, cavitation or additional pathology was graded as the percentage of the affected lung [33]. Finally, the total percentage of the lungs affected by any pathology was estimated.

### Preparation of blood samples

Peripheral blood mononuclear cells (PBMC) and plasma were separated from whole blood by Ficoll-Paque^TM^ PLUS (GE Healthcare, Uppsala, Sweden) density gradient centrifugation. Plasma samples were stored at -20°C for molecular analysis, while isolated PBMCs were washed and resuspended in culture medium (RPMI-1640 supplemented with 10% autologous plasma, 1% L-glutamine, 1% sodium pyruvate and 1% penicillin-streptomycin (Gibco, Grand Island, NY, USA)) and plated in two separate 4-well cell culture plates (NUNC, Roskilde, Denmark). One cell culture plate was used for mRNA isolation and analyses of LL-37 peptide, while the other plate was used for assessment of *Mtb* killing mediated by patient’s MDM.

After three days incubation of PBMCs in 4-well plates, the culture supernatants containing non-adherent cells were removed and centrifuged to collect the clear supernatant or the extracellular fluid (ECF) of PBMCs. Flow cytometry were used to determine that >80% of non-adherent cells in the plates were CD3^+^ and CD19^+^ lymphocytes while >90% of adherent cells were monocytes (CD14^+^ MDM). The cell pellet of non-adherent lymphocytes were treated with 0.1% saponin (Sigma-Aldrich, Steinheim, Germany) for formation of reversible pores in the cell membrane to release their intracellular content, which was collected as intracellular fluid (ICF) after centrifugation. Next, the adherent MDM were harvested using a cell scraper and treated similarly with saponin, the ICF was collected and stored until further analysis. RNAlater (Qiagen GmbH, Hilden, Germany) was added to the MDM cell pellets to prepare for mRNA isolation and subsequent analysis of LL-37 mRNA content.

### Assessment of LL-37 peptide and level of mRNA encoding the LL-37 peptide

LL-37 peptide levels were measured by ELISA in ICF of MDM and non-adherent lymphocytes and in ECF of PBMC. A standard curve of the ELISA was generated from synthetic LL-37 (Innovagen, Lund, Sweden). Polystyrene microtiter plates (Maxisorp by NUNC, Naperville, IL, USA) were coated with monoclonal anti-LL-37 (5 μg/ml) [34] in carbonate buffer (15 mM sodium carbonate, 35 mM sodium bicarbonate and 0.02% sodium azide [pH 9.6]) and incubated overnight at 4°C. Non-specific binding was blocked after washing with 0.1% gelatin in Tris-buffered saline for 1 hour at RT. Next, patient samples and the diluted standard were added and incubated overnight at 4°C. After sequential incubations with biotinylated rabbit anti-LL-37 (1 μg/mL) (Innovagen) and Streptavidin-alkaline phosphatase conjugate (Chemicon, Melbourne, Australia) for 2h each at RT, the reaction was developed using 4-methyl-umbelliferyl phosphate as the substrate (Molecular Probes, Leiden, The Netherlands). Fluorescence was measured at an excitation wavelength of 360 nm and emission wavelength of 450 nm.

mRNA was extracted from MDM and NAL using the RNeasy Mini kit as described by the manufacturer (Qiagen GmbH). mRNA was reverse-transcribed using Bio-Rad CFX 1000, (Hercules, CA, USA) and cDNA was synthesized using Superscript III First-Strand Synthesis System (Invitrogen, Grand Island, NY, USA). The relative expression of mRNA encoding LL-37 peptide compared to the housekeeping gene 18S rRNA was measured by real-time quantitative RT-PCR using the CFX96 Real-Time PCR Detection Systems (Bio-Rad,) and the 18S rRNA-housekeeping gene kit (Applied Biosystems, Foster City, CA, USA). The sequences of forward and reverse primers for mRNA encoding the LL-37 peptide were 5´-TCACCAGAGGATTGTGACTTCAA-3´ and 5´-TGAGGGTCACTGTCCCCATAC-3´, respectively (Primer Express; Applied Biosystems). The results were analyzed by using a relative standard method [35].

### Effector function of MDM

To determine the capacity of *Mtb*-infected MDM in the killing of intracellular *Mtb*, a bactericidal assay was performed as an effector function test of MDM. MDM obtained from patients at baseline, week 4, 8 and 12 were used for this experiment. MDM were seeded in culture plates and infected with virulent *Mtb* strain H37Rv (Tuberculosis Research Center, Chennai, India) at a multiplicity of infection (MOI) of 25:1 in culture medium without antibiotics [26] after testing 3 different ratios (10:1; 25:1 and 50:1) for an optimal MOI. After 2 hours, the culture plates were washed with warm RPMI to remove the extracellular bacteria. Infected MDM were cultured for three additional days in a medium with autologous plasma and antibiotics (penicillin-streptomycin, amphotericin B) (Gibco, Grand Island, NY, USA). Thereafter, the cells were lysed with 0.3% saponin-PBS followed by vigorous pipetting to collect viable intracellular *Mtb*. The cell lysates were cultured on Middle-Brook 7H11 agar medium (Becton Dickinson, Sparks, MD, USA) for 21–28 days at 37°C and bacterial viability was calculated by counting colony forming units (CFU). The level of MDM-mediated killing at day 0, before initiation of any intervention, served as control for all groups. A ‘relative CFU count’ was calculated for each time point by normalizing the data to the CFU of the *Mtb* inoculum [11]. A cut-off of 0.1 was considered as zero because zero values are not obtained when data is normalized with inoculum data; there was always a residual *Mtb* count.

### Assessment of vitamin D, calcium and C-Reactive Peptide (CRP) in plasma

Plasma levels of 25(OH)D_3_ was estimated by an electrochemiluminescence immunoassay analyzed on an automated Roche immunoassay analyzer (Cobas e601) using a Vitamin D_3_ Kit (Roche Diagnostics GmbH, Mannheim, Germany). This method is standardized against standard LC-MS/MS [36] which in turn has been standardized to the NIST standard [37]. Results were determined using a calibration curve that was generated by a Vitamin D_3_ CalSet (Roche). According to the manufacturer, this assay shows 100% and 92% cross-reactivity with 25-OH vitamin D_3_ and 25-OH vitamin D_2_, respectively. Commercial quality control material Elecsys PreciControl Varia (Roche) was used as an internal quality control. The Laboratory also participates in External Quality Assurance Schemes [38, 39].

Calcium and albumin were measured in plasma by two colorimetric assays, Calcium Gen.2 and ALB plus kit (Roche Diagnostics) respectively, while CRP was determined by an immunoturbidometric method (Roche Diagnostics). All results were obtained using an automated clinical chemistry analyzer (Hitachi 902, Roche diagnostics). Quality control material, Precinorm U and Precipath U (for calcium and albumin) as well as Precinorm Protein and Precipath Protein (for CRP) from Roche diagnostics, were used as internal quality controls. Plasma calcium concentrations were adjusted to plasma albumin. Normal serum calcium (albumin adjusted) range is 8.6 to 10.5 mg/dL. Hypercalcaemia was defined at serum albumin-adjusted Ca >10.5 mg/dL.

Concentration of PBA in plasma could not be measured due to lack of access to an appropriate method.

### Statistical analysis

Statistical analysis was performed using IBM SPSS Statistics 20.0 and Stata 13 (StataCorp, College Station, Texas, USA). Data not normally distributed were log transformed that included CRP, LL-37 peptide expression in MDM and lymphocytes as well as plasma 25(OH)D_3_ levels. A p-value of ≤0·05 was considered significant. The primary analysis of the outcomes of interest was performed by modified intention-to-treat (ITT) for up to week 12 and per-protocol analysis for week 24. Efficacy was assessed by modified ITT that excluded patients who had a negative sputum culture at baseline. Outcome variables were reported as means, with 95% confidence intervals (CI) or standard deviations when continuous, and categorical variables were reported as numbers with percentages. The Chi-square test was used to compare the proportion of patients who became sputum culture negative at week 4 and 8 compared to placebo. Effect size was estimated as difference in proportion of outcome of interest (sputum culture conversion and clinical endpoints) among treatment groups and strength of effect size was examined using Odds Ratio (OR) generated by multivariable logistic regression model. In the model, placebo was used as reference. A mixed model ANCOVA was used to follow-up outcome values as dependent variable and treatment arm as independent variable, to investigate effectiveness of TB scores, leukocyte and monocyte counts, concentrations of hemoglobin, ESR, CRP, calcium, LL-37 and 25(OH)D_3_ between the treatment arms and placebo. Least significant difference (LSD) was used for multiple comparison test of means of targeted parameters across the treatment arms. We initially investigated potential treatment and covariate interactions and adjusted for those covariates that were significantly associated (by 5% or more) with the outcomes. The covariates examined in the analyses were age, sex, duration of illness, BCG vaccination status, and history of contact with active TB cases. Analyses of time to sputum smear conversion (at week 0–4, 6, 8, 10, 12) and MDM-mediated killing of *Mtb* (at week 0, 4, 8 and 12) were conducted using Kaplan-Meier curves and log-rank (Mental–Cox) test. The Tukey-Kramer method was used to adjust for multiple pair-wise comparisons since the analyses included more than two groups.

## Results

### Enrollment procedure and demography

**Fig 1:**
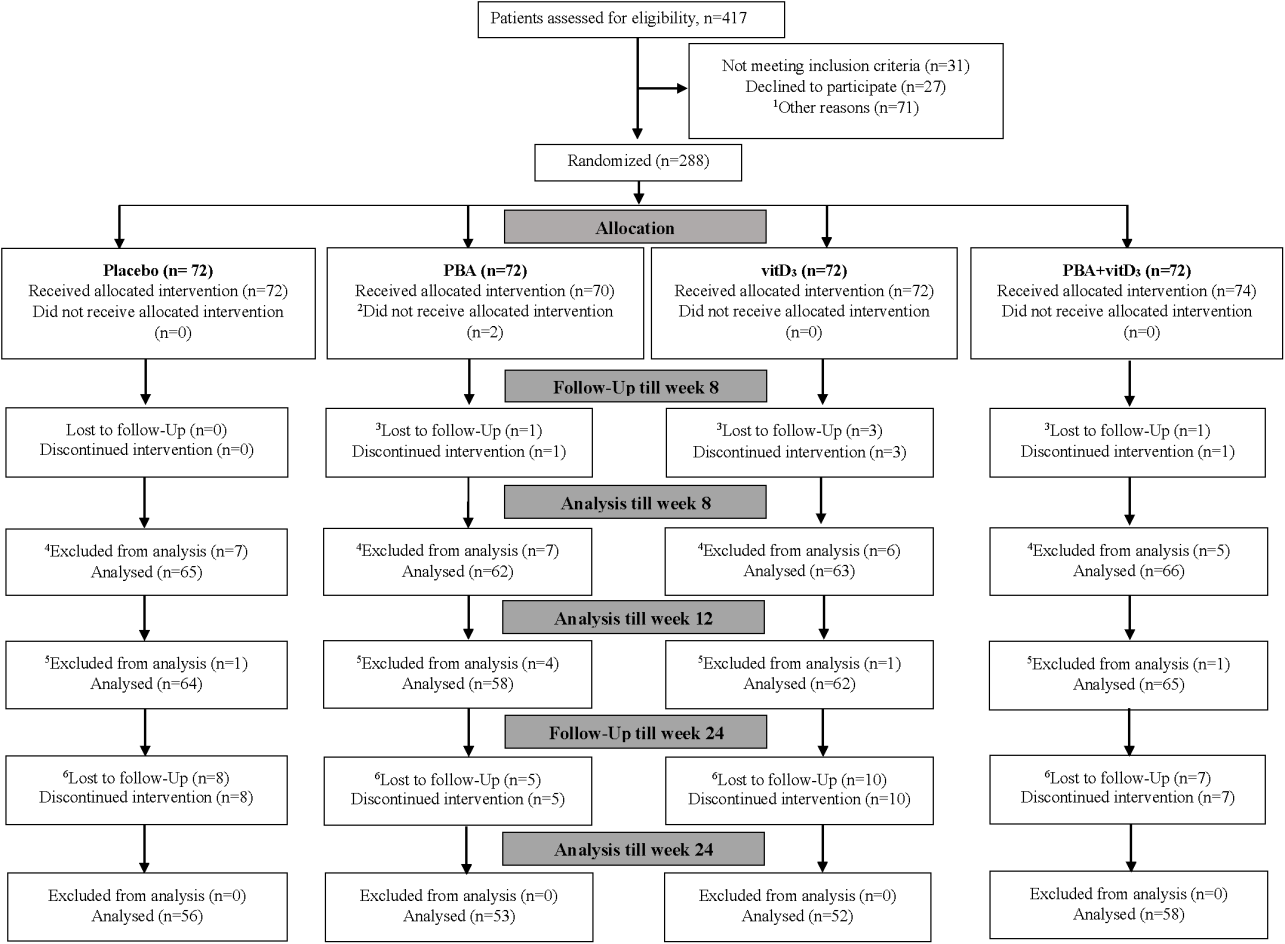
Consort flow diagram of patients with tuberculosis, from screening to analysis. PBA, Phenylbutyrate; vitD_3_, vitamin D_3_; ^1^Other reasons for not randomizing include living outside Dhaka, difficult to continue in the trial due to job- and academic activity-related problems. ^2^At base line two participants in the PBA-group did not receive allocation as they refused to continue in the study just after enrollment. ^3^There were five dropouts between enrollment and week 8, due to migration to other cities, could not be contacted or refused to continue since they moved from Dhaka to their respective village homes in the country side. ^4^Excluded from analysis: 28 patients were culture negative at baseline, among them, 3 are included in the above 5 dropouts. ^5^Excluded from analysis: seven patients had multidrug resistant tuberculosis (MDR TB) unevenly distributed among the treatment arms. ^6^There were thirty patients who discontinued the intervention between week 12 to 24, due to migration, pilgrimage, sent to jail, could not be contacted via phone or when visits to respective homes were made, refused to come to Dhaka for follow-up visits since they moved to their village homes.

A total of n = 417 sputum smear-positive pulmonary TB patients were screened for eligibility between December 14, 2010, and December 26, 2013. Of these, n = 31 patients did not meet the inclusion criteria, n = 27 patients did not provide consent and n = 71 patients were excluded due to the various reasons described in the flowchart in **Fig 1**. A total of n = 288 TB patients were finally enrolled in the study and randomized into the four treatment arms. Two patients in the PBA-group refused to continue in the study just after enrollment and did not receive the allocated adjunctive therapy. Five patients declined to continue in the study after 4 to 6 weeks since they migrated from Dhaka; 30 dropped out from the study after completing 12 weeks treatment. Of the 288 sputum smear positive patients, 28 patients had *Mtb* negative cultures at baseline; most likely due to non-tuberculosis mycobacteria (NTM) and 7 patients were diagnosed with MDR-TB. Thus, 7 MDR-TB cases, 28 culture negative cases (3 patients included in the dropout cases between 4–6 weeks) were excluded from the analysis for modified ITT analysis (**Fig 1**). In total 219 patients completed the trial by the follow-up visits at week 24 and formed the per-protocol population.

### Baseline characteristics

Male patients were slightly higher in numbers than female patients (ratio 1.6:1) in each group (**Table 1**). History of contact with active TB cases was less than 33%. About 60–74% patients were deficient in vitamin D status, and only 7–13% patients had sufficient status. An important feature in TB patients was the very low body weight at baseline with minimum increase after 2 or 6 months’ therapy. No differences were found in baseline characteristics when randomized patients were compared to potentially eligible patients who declined or had to be excluded from the study (**S2 Table**).

**Table 1:** Baseline characteristics of patients with freshly diagnosed pulmonary tuberculosis in the four treatment groups.

Features	Placebo (n = 72)	PBA (n = 72)	vitD_3_ (n = 72)	PBA+vitD_3_ (n = 72)
**Intention-to-treat group, n = 288**				
Gender (Males) (Number, %)	44(61%)	44(61%)	44(61%)	45(62·5%)
History of contacts (Number, %)				
Male	10(22.7%)	12(27.3%)	9(20.5%)	12(26.7%)
Female	13(46.4%)	8(28.6%)	10(35.7%)	12(44.4%)
BCG given (Number, %)				
Male	33(75.0%)	24(54.5%)	35(79.5%)	32(77.1%)
Female	13(46.4%)	20(71.4%)	20(71.4%)	21(77.8%)
Age, years (Mean±SD)	26.7±8.1	26.8±7.3	28.1±9.9	26.8±6.9
Weight, kg (Mean±SD)				
Male	46.3±6.6	48.9±6.5	47.9±8.4	46.6±6.6
Female	39.8±7.8	37.0±5.7	38.4±7.6	39.8±7.8
Tuberculin skin test done	7(2.4%)	12(4.2%)	8(2.8%)	9(3.1%)
Duration of illness, days (Mean±SD)				
Male	50.9±26.8	48.8±20.6	55.1±2 6.5	53.6±28.7
Female	51.9±23.5	53.6±19.9	51.9±26.5	47.2±22.9
ESR, mm 1^st^ hr (Mean±SD)	60.2±34.9	56.9±32.5	54.0±31.1	56.8**±**33.2
Hb, gm/dl (Mean±SD)	11.6±1.6	11.5±1.7	11.3±1.9	11.7±1.8
WBC, 1x10^3^/cmm (Mean±SD)	10.56±2.63	9.99±2.16	10.93±2.88	11.36±3.21
**Per-protocol group, n = 249**				
**Vitamin D status** ^**#**^	**(n = 64)**	**(n = 58)**	**(n = 62)**	**(n = 65)**
Vitamin D nmol/L	28.1±16.2	23.8±14.8	28.0±17.5	26.8±16.3
Deficient, <30 nmol/L	40(62.5%)	41(70.7%)	46(74.2%)	39(60.0%)
Insufficient, 30–50 nmol/L	19(29.7%)	13(22.4%)	8(12.9%)	20(30.8%)
Sufficient, >50 nmol/L	5(7.8%)	4(6.9%)	8(12.9)	6(9.2%)

Data is presented as mean ± standard deviation or number with percentage in parentheses. BCG, *Bacillus Calmette–Guérin*.; ESR, erythrocyte sedimentation rate; Hb, hemoglobin; WBC, white blood cells. History of contacts, BCG given and Tuberculin skin test done are dichotomous variable and age, weight, duration of illness, ESR, Hb, WBC and vitamin D status are continuous variable.

### Adverse events and serious adverse events

There were no differences in the occurrence and distribution of most of the AEs between the study arms (**Table 2**). The occurrence of vomiting was significantly lower in the PBA-, vitD_3_- and PBA+vitD_3_- groups compared to placebo (*p* = 0.055, 0.022 and 0.044 respectively). The most common AEs were anemia (61.9%), anorexia (41.2%), joint pain or arthralgia (28.5%) followed by chest pain (18.7%), vomiting (9.5%) and body ache (7.4%). Arthralgia increased in all study groups after initiation of anti-TB therapy and was mostly manifested at week 8 (average 54%) but declined until week 12 (average 31%). No cases of hypercalcaemia were noted in the trial. However, hypocalcaemia was common among the patients in the 4 groups ranging from 26% to 48% at baseline (**Table 5**) without any significant changes during the study period.

**Table 2:** Distribution of major types of adverse effects observed during the treatment of tuberculosis by different groups at week 4 (modified intention-to-treat analysis, n = 284).

Types of adverse effects	Placebo n = 72	PBA n = 69	vitD_3_ n = 71	PBA+vitD_3_ n = 72
Anemia	42(58.3%)	46(64.7%)	42(59.2%)	46(63.9%)
Anorexia	33(45.8%)	25(36.2%)	28(39.4%)	31(43.1%)
Joint pain	20(27.8%)	18(26.1%)	26(36.6%)	17(23.6%)
Chest pain	16(22.2%)	10(15.5%)	15(21.1%)	12(16.7%)
Nausea and or vomiting	13(18.1%)^a^	5(7.2%)^b^	4(5.6%)^b^	5(6.9%)^b^
Body ache	6(8.3%)	4(5.8%)	4(5.6%)	7(9.7%)
General weakness	4(5.6%)	2(2.9%)	7(9.9%)	8(11.1%)
Itching or skin irritation	3(4.2%)	2(2.9%)	1(1.4%)	2(2.8%)
Dyspepsia	3(4.2%)	4(5.8%)	1(1.4%)	3(4.2%)
Vertigo	1(1.4%)	3(4.3%)	2(2.8%)	2(2.8%)
Abdominal pain	2(2.8%)	3(4.3%)	1(1.4%)	4(5.6%)

**Note**. Data is presented as numbers with percentage in parentheses. Two patients in the PBA group did not receive allocation and one dropped out from the study between 0–3 weeks. One patient in the vitD_3_ group dropped out from the study between 0–3 weeks.

^a,b^Different superscripts in a row show significant difference between the groups. Significance *p*≤0.05.

There were four cases of SAEs that included hospitalization; one patient had severe vomiting with breathing difficulties, two patients experienced severe reactions of anti-TB treatment including elevated levels of serum glutamic pyruvic transaminase (SGPT), and one patient had severe loin pain due to urinary tract infection (**Table 3**). The patients with SAEs were advised complete rest without interrupting anti-TB therapy. None of the SAEs had any connection to the study drugs (vitD_3_ or PBA), as reviewed by the DSMB.

**Table 3:** Symptoms of patients with serious adverse events.

Patient	Sex	Symptoms of serious adverse events	Treatment arms
001	Female	Nausea, profuse vomiting, and breathing difficulties	PBA+vitD_3_
002	Female	Severe abdominal pain, nausea, vomiting, left loin pain	Placebo
003	Female	Body ache, vomiting, generalized weakness, elevated SGPT	PBA
004	Male	Nausea, vomiting and low grade fever, elevated SGPT	PBA

Note. SGPT, serum glutamic pyruvic transaminase

### Primary endpoint 1: Microbiological outcome

Excluding 28 culture negative cases and 7 MDR-TB cases, the number of patients who completed week 4 was 249, which constituted the modified ITT population for analysis of primary endpoint 1. Chi-square test was applied to compare the proportion of patients between intervention groups and placebo. The proportion of patients being culture-negative at week 4 were higher in the PBA+vitD_3_-group (71%; 46/65) (*p* = 0.001) and the vitD_3_-group (64.4%; 38/62) (*p* = 0.032) compared to the placebo-group (43.7%; 27/64). In the PBA-group, 46.6% (27/58) were culture-negative at week 4 (*p* = 0.62). The proportion of patients being culture-negative at week 8 were higher in the vitD_3_-group (98.4%; 61/62) (*p* = 0.032) and the PBA+vitD_3_-group (95.4%; 62/65) (*p* = 0.179) compared to the placebo-group (89.1%; 57/64). In the PBA-group, 91.4% (53/58) were culture-negative at week 8 (*p* = 0.668).

**Fig 2:**
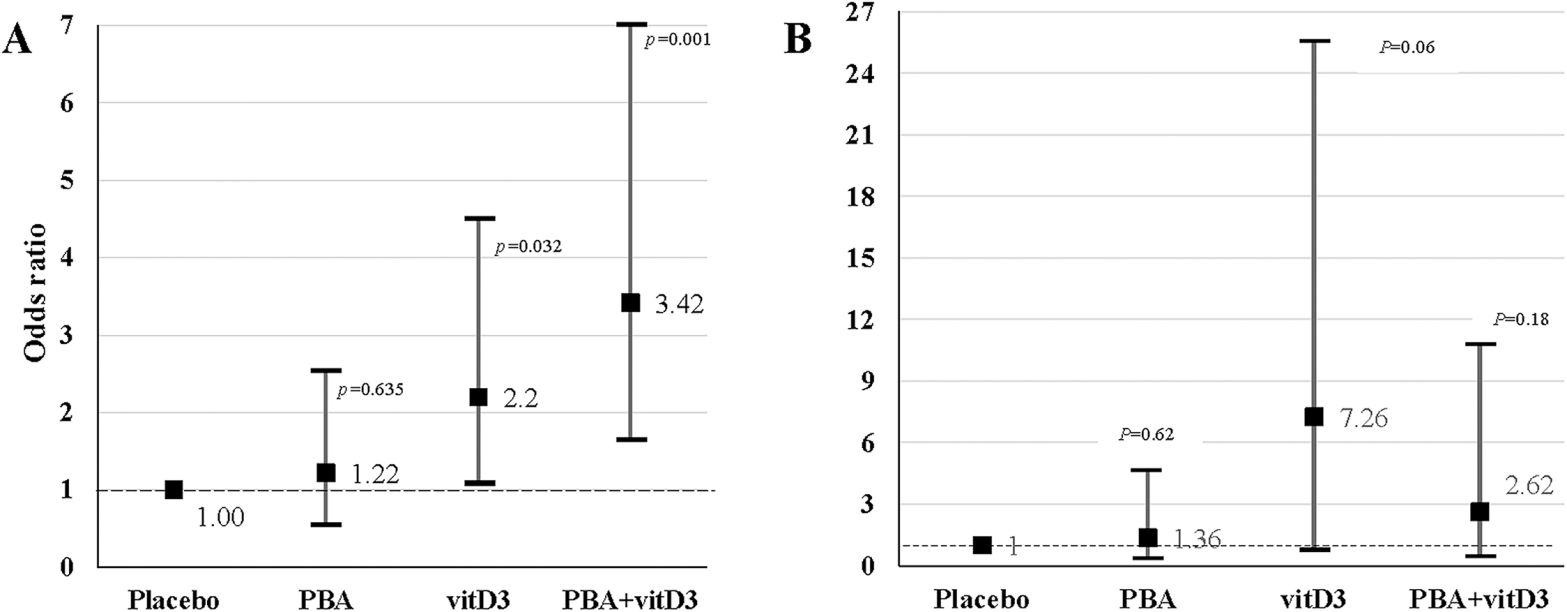
Multivariable logistic regression model was used to estimate the effect of adjunct therapy on the sputum culture conversion (culture negative) and sputum smear conversion at week 4 and 8. Points show the age- and sex-adjusted odds ratio (OR) values, and vertical lines delineate 95% confidence intervals. Adjusted OR is shown for four treatment groups (PBA, vitD_3_ and PBA+vitD_3_) at week 4 and 8 vs. placebo group. (A) The odds of sputum culture being negative at week 4 was 3.42 times higher in the PBA+vitD_3_-group (95% Confidence interval (CI), 1.64–7.15) and 2.20 times higher in vitD_3_-group (95% CI, 1.07–4.51) compared to the placebo-group. (B) The odds of sputum culture being negative at week 8 was 7.26 times higher in the vitD_3_-group (95% CI, 0.06–25.5), 2.62 times higher in PBA+vitD3-group (95% CI, 0.64–10.72) and 1.36 times higher in the PBA-group (95% CI, 0.40–4.59) compared to the placebo-group.

The odds ratio of sputum culture conversion was estimated by using multivariable logistic regression adjusting for age and sex. The odds of sputum culture being negative at week 4 was 3.42 times higher in the PBA+vitD_3_-group (95% Confidence interval (CI), 1.64–7.15, *p* = 0.001), 2.20 times higher in vitD_3_-group (95% CI, 1.07–4.51, *p* = 0.032) and only 1.22 times higher in the PBA-group (95% CI, 0.59–2.53, *p* = 0.587) compared to the placebo-group (**Fig 2A**). The odds of sputum culture being negative at week 8 was 7.26 times higher in the vitD3-group (95% CI, 0.90–25.50, *p* = 0.062), 2.62 times higher in PBA+vitD3-group (95% CI, 0.64–10.12, *p* = 0.181) but only 1.36 times higher in the PBA-group (95% CI, 0.40–4.59, *p* = 0.625) compared to the placebo-group (**Fig 2B**).

### Primary endpoint 2: Clinical endpoints at week 8

The odds ratio of major clinical endpoints was estimated by using multivariable logistic regression adjusting for age and sex. There were 249 patients who completed week 8, which formed the modified ITT population for analysis of primary endpoint 2. Only patients in the PBA-group had higher recovery from fever at week 2 compared to placebo (**Table 4**). The odds of a significant increase in weight gain was lower in the vitD_3_-group (*p* = 0.03) and PBA+vitD_3_ (*p* = 0.066) at week 4 compared to placebo.

**Table 4:** Odds ratio for major clinical endpoints in TB patients at various intervals after initiation of anti-TB treatment and adjunctive therapy (modified intention-to-treat analysis).

	Fever	Cough	Weight gain	Chest x-ray
Week 2	Adjusted OR (95% CI)	Adjusted OR (95% CI)	Adjusted OR (95% CI)	Adjusted OR (95% CI)
Placebo	Reference	Reference	Reference	
PBA	1.93(0.93–4.03)	1.25(0.47–3.36)	0.79(0.38–1.63)	
vitD_3_	1.21(0.58–2.53)	0.31(0.08–1.19)	0.94(0.46–1.90)	
PBA+vitD_3_	1.30(0.63–2.68)	0.60(0.20–1.81)	0.67(0.33–1.36)	
**Week 4**				
Placebo	Reference	Reference	Reference	
PBA	1.14(0.55–2.33)	1.47(0.66–3.29)	0.68(0.33–1.39)	
vitD_3_	0.97(0.46–1.89)	0.94(0.41–2.17)	0.46(0.23–0.94)	
PBA+vitD_3_	1.39(0.69–2.81)	0.58(0.24–1.42)	0.52(0.26–1.05)	
**Week 8**				
Placebo	Reference	Reference	Reference	Reference
PBA	1.05(0.49–2.22)	1.56(0.75–3.20)	0.96(0.47–1.98)	1.50(0.65–3.44)
vitD_3_	1.37(0.65–2.92)	1.65(0.82–3.66)	0.93(0.46–1.89)	0.87(0.35–2.18)
PBA+vitD_3_	1.57(0.74–3.35)	1.32(0.66–2.66)	0.80(0.39–1.61)	1.55(0.67–3.56)

Note. The decrease in fever to normal temperature, disappearance of cough, reduction of lung involvement (as judged by chest x-ray) and increase in weight at various intervals were considered as clinical endpoints.

### Secondary outcomes

#### Sputum smear conversion

**Fig 3:**
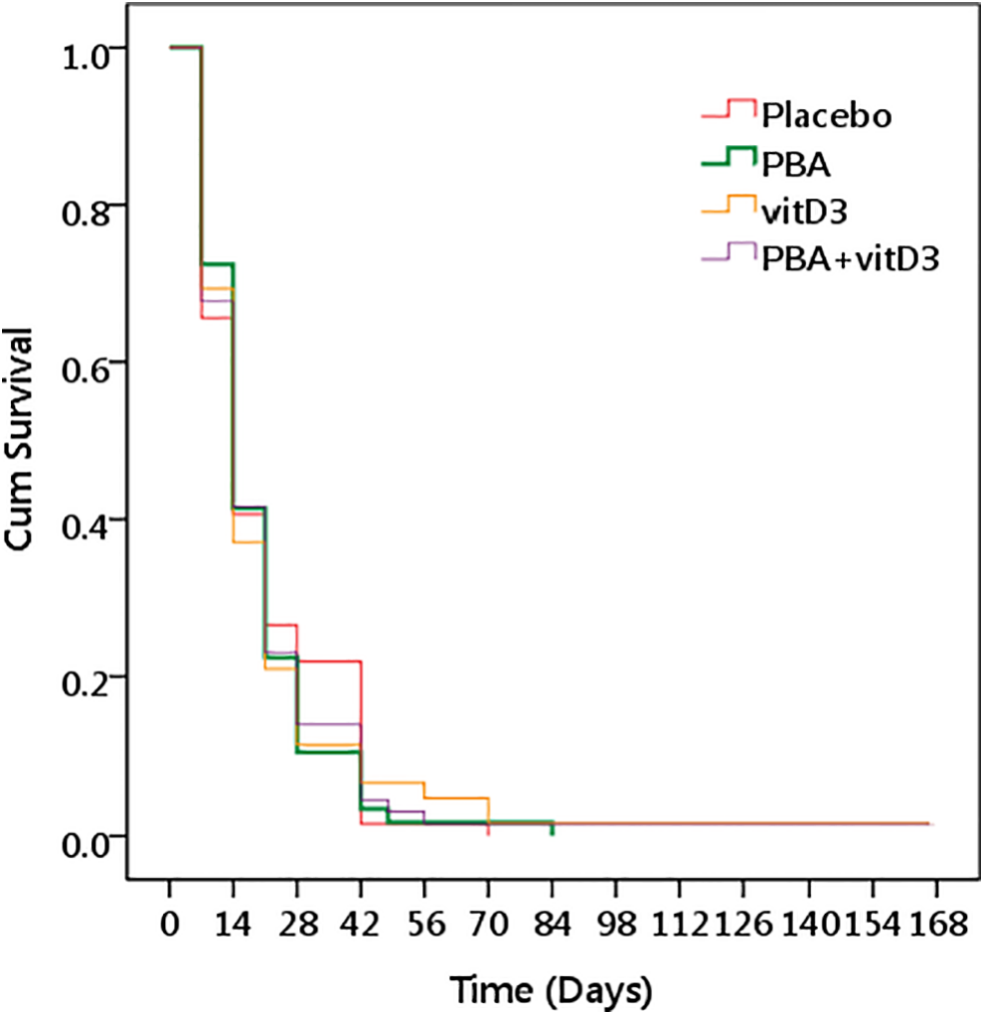
Kaplan Meier survival plot for impact of the different interventions on time to sputum smear becoming negative. The log rank analysis showed no significant differences between the placebo and the intervention groups (log rank 0.228, *p* = 0.973).

Comparison of time to sputum smear conversion (sputum smear becoming negative) for the different intervention groups compared to placebo was performed using Log Rank test. No significant differences were obtained between the placebo and intervention groups (log rank 0.228, *p* = 0.973) (**Fig 3**).

#### Vitamin D status and blood chemistry

**Fig 4:**
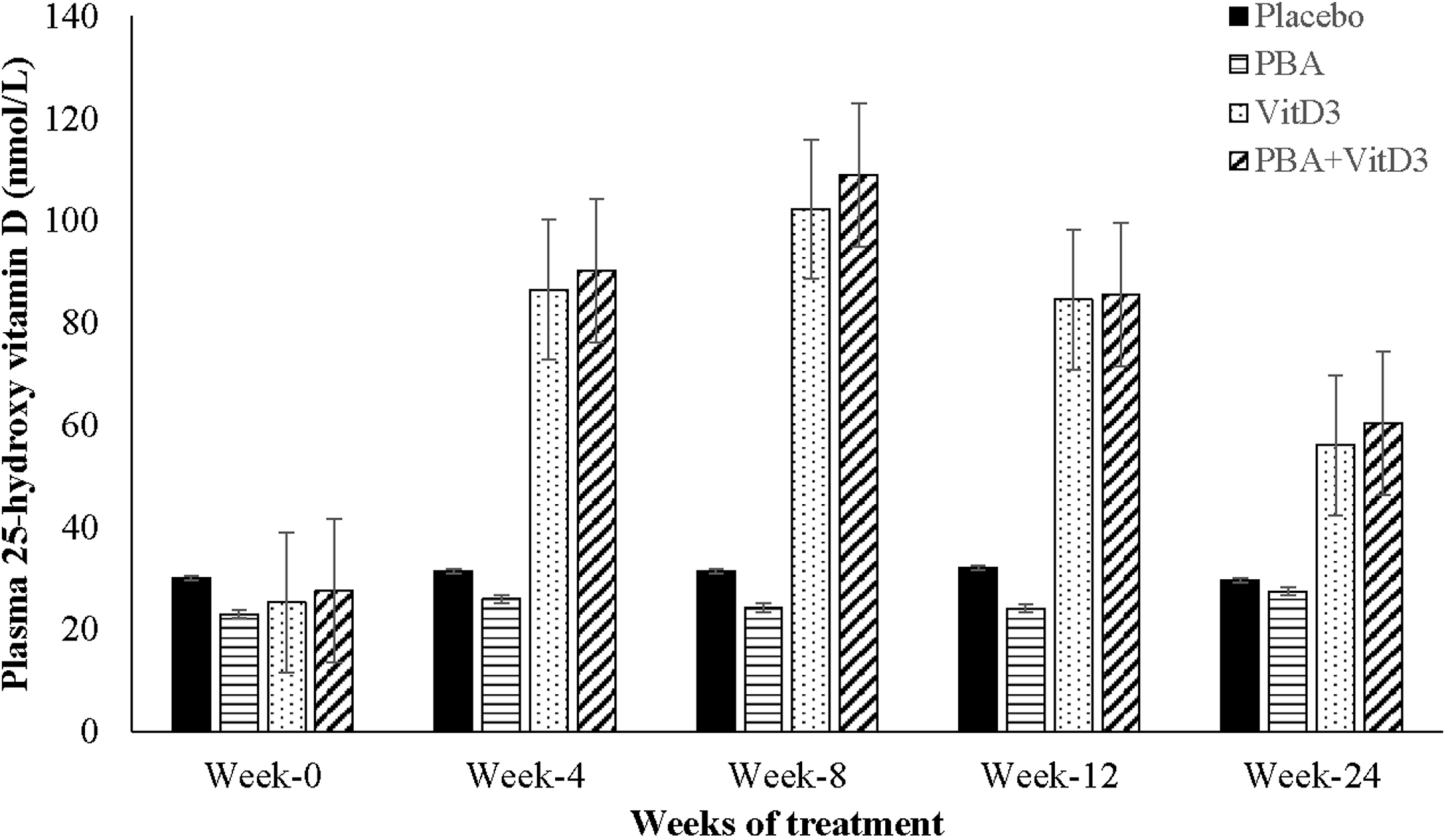
Plasma concentration of 25-hydroxyvitamin D_3_ at baseline, week 4, 8 and 12 after initiation of treatment in TB patients in the four intervention arms. The groups receiving vitD_3_ supplementation (vitD_3_ and PBA+vitD_3_-groups) exhibited significantly higher concentrations of plasma 25-hydroxyvitamin D_3_ at week 4, 8 and 12 intervals compared to placebo after initiation of therapy (p<0.000 for all).

The standard classification proposed by the Institute of Medicine (IOM) was followed as sufficient vitamin D status, >50 nmol/L; insufficient status, 30–50 nmol/L; and deficient status, below 30 nmol/L [40]. There were no differences in plasma 25(OH)D_3_ levels between the interventions and placebo-groups at baseline when 67% of the patients exhibited deficient status of 25(OH)D_3_, 24% had insufficient and 9% had sufficient concentrations of 25(OH)D_3_ (**Fig 4**; **S3 Table**). Accordingly, vitD_3_ supplementation was warranted in this study population. Since there were interaction effects of age, sex, history of contact and BCG status on plasma 25(OH)D_3_ levels, these were used as covariates in the ANCOVA model. Administration of vitD_3_ to the TB patients in the presence or absence of PBA (vitD_3_-supplemented group) from week 0 to 8, resulted in significantly elevated levels of 25(OH)D_3_ at week 4, 8, 12 and 24 compared to the non-vitD_3_ supplemented groups (PBA and placebo), which remained significantly higher at week 24 (*p*<0.001 for all) (**Fig 4**). At week 8, 94%-100% patient among the vitD_3_-supplemented group attained sufficient vitamin D status as opposed to 2%-14% in the non-supplemented group (**S3 Table**). About 63–67% of vitD_3_-supplemented group and 11–15% of non-vitD_3_ supplemented group retained the sufficient levels of plasma 25(OH)D_3_ (> 50 nmol/L) at week 24.

No cases of hypercalcemia were obtained after adjunct therapy; however, prevalence of hypocalcemia was common in all groups. Hypocalcemia was defined as concentration of adjusted plasma calcium <8.6 mg/dL (**Table 5**). During the study period, no major effects of adjunct therapy was observed with regards to body weight, albumin-adjusted mean plasma calcium, CRP, ESR, hemoglobin, total leukocytes and lymphocyte counts (**Table 5**). From 13 patients, hematological reports were not available at week 24. Four weeks after initiation of adjunct therapy, neutrophil counts were lower in the PBA and PBA+vitD_3_-groups compared to the placebo-group (*p* = 0.06 and *p* = 0.058, respectively). At week 24 monocytes counts were significantly lower in the PBA+vitD_3_ group compared to placebo (*p* = 0.047).

**Table 5:** Body weight and concentrations of blood parameters in patients with tuberculosis at various intervals after initiation of anti-TB treatment and adjunctive therapy (modified intention-to-treat analysis).

	Intervals	Placebo n = 64	PBA n = 58	vitD_3_ n = 62	PBA+vitD_3_ n = 65
CRP, μg/dL	Week 0	32.14±0.37	25.06±0.37	26.18±0.37	34.51±0.37
	Week 4	10.67±0.53	7.36±0.53	8.61±0.53	9.91±0.54
	Week 8	5.80±0.60	4.88±0.60	5.68±0.60	5.82±0.60
	Week 12	3.12±0.58	2.98±0.57	3.06±0.57	3.06±0.57
Adjusted Ca, mg/dL	Week 0	8.65±0.59	8.65±0.56	8.82±0.55	8.67±0.58
	Week 4	8.61±0.57	8.56±0.61	8.74±0.69	8.59±0.65
	Week 8	8.46±0.67	8.37±0.61	8.57±0.61	8.54±0.59
	Week 12	8.50±0.57	8.55±0.66	8.69±0.70	8.61±0.57
Hypocalcaemia	Week 0	29 (45.3%)	25 (41.7%)	16 (26.2%)	31 (47.7%)
	Week 4	31 (48.4%)	28 (46.7%)	27 (44.3%)	31 (47.7%)
	Week 8	33 (51.6%)	38 (63.3%)	34 (55.7%)	32 (49.2%)
	Week 12	31 (48.4%)	26 (43.3%)	22 (36.1%)	32 (49.2%)
Hemoglobin, g/dL	Week 0	11.62±1.60	11.68±1.72	11.03±1.80	11.76±1.85
	Week 4	12.08±1.80	12.36±1.77	11.95±1.84	12.16±2.91
	Week 8	12.41±1.58	12.36±1.89	12.29±1.89	12.29±1.81
	Week 24^#^	13.41±1.71	13.53±1.92	13.16±1.96	13.29±1.87
ESR, mm 1^st^ hr	Week 0	60.4±35.3	57.4±31.9	49.8±29.2	57.8±34.5
	Week 4	40.0±27.2	37.7±28.9	41.3±25.4	41.6±30.3
	Week 8	36.3±22.2	35.4±27.0	33.5±22.0	32.5±20.1
	Week 24^#^	23.0±18.0	20.9±15.6	21.1±17.1	25.2±17.4
Total leukocyte,	Week 0	10.59±2.66	9.85±2.67	10.87±2.66	11.33±2.66
1x10^3^/cmm	Week 4	9.55±2.54	9.26±2.55	9.89±2.54	9.54±2.54
	Week 8	8.99±2.20	8.85±2.21	8.50±2.20	8.41±2.21
	Week 24^#^	8.24±2.17	7.80±2.18	8.56±2.18	7.88±2.18
Lymphocytes %	Week 0	23.65±7.88	24.85±7.92	24.18±7.90	24.36±7.90
	Week 4	25.81±8.93	28.73±8.97	28.11±8.94	28.43±8.94
	Week 8	28.73±9.16	29.43±9.20	30.92±9.17	30.14±9.17
	Week 24^#^	34.78±12.04	33.47±12.08	33.78±12.04	35.29±12.04
Neutrophils %	Week 0	70.30±8.78	68.41±8.82	69.34±8.79	69.81±8.80
	Week 4	67.54±10.13	63.80±10.18	64.65±10.15	63.83±10.15
	Week 8	63.70±10.10	63.05±10.14	61.35±10.12	61.74±10.12
	Week 24^#^	55.39±12.37	56.54±12.42	55.70±12.38	54.86±12.39
Monocyte, %	Week 0	3.34±2.38	3.90±3.43	3.39±2.37	3.57±2.31
	Week 4	3.51±2.38	3.93±2.79	3.50±2.33	3.46±2.45
	Week 8	3.52±2.36	3.98±3.17	4.32±2.54	4.21±2.96
	Week 24^#^	5.62±3.87^a^	5.31±2.81	4.93±2.84	4.46±2.38^b^
Body weight, Kg	Week 0	43.7±7.4	44.4±9.1	44.2±9.4	44.1±7.7
	Week 4	44.2±7.3	45.1±9.2	45.2±8.9	44.9±8.0
	Week 8	44.9±7.6	45.8±9.3	46.3±9.5	45.6±8.1
	Week 12	45.6±7.7	46.5±9.3	47.0±9.5	46.3±7.9

Data is presented as mean ± standard deviation or numbers with percentage in parentheses. ESR, erythrocyte sedimentation rate; CRP, C-reactive protein. In a row, different superscripts show significant difference between the groups at a given time point.

^#^At week 24, sample size in four groups were: placebo, n = 54; PBA, n = 49, vitD_3_, n = 49; PBA+vitD_3_, n = 54. From 13 patients, hematological reports were not available. Hypocalcemia was defined as concentration of adjusted plasma calcium <8.5 mg/dL. Statistical analyses were done by ANCOVA adjusting for covariates. There were interaction effects of age, sex, contact history and BCG status on ESR and monocyte counts and for the rest of the data interactions were obtained with age and sex only. P is significant when *p*≤0.05.

### Effects of adjunct therapy on LL-37 expression in immune cells

ANCOVA model was applied to compare the difference in means of LL-37 peptide/mRNA concentrations of the 3 intervention groups with that of the placebo at various time intervals. Concentration of LL-37 peptide was measured in 3 different cell types, thus analysis was performed for each cell type. Age, sex, contact history and BCG status were used as covariates for analyzing LL-37 peptide in lymphocytes and PBMC, and LL-37 mRNA in MDM, while only age and contact history were adjusted for LL-37 peptide in MDM.

**Fig 5:**
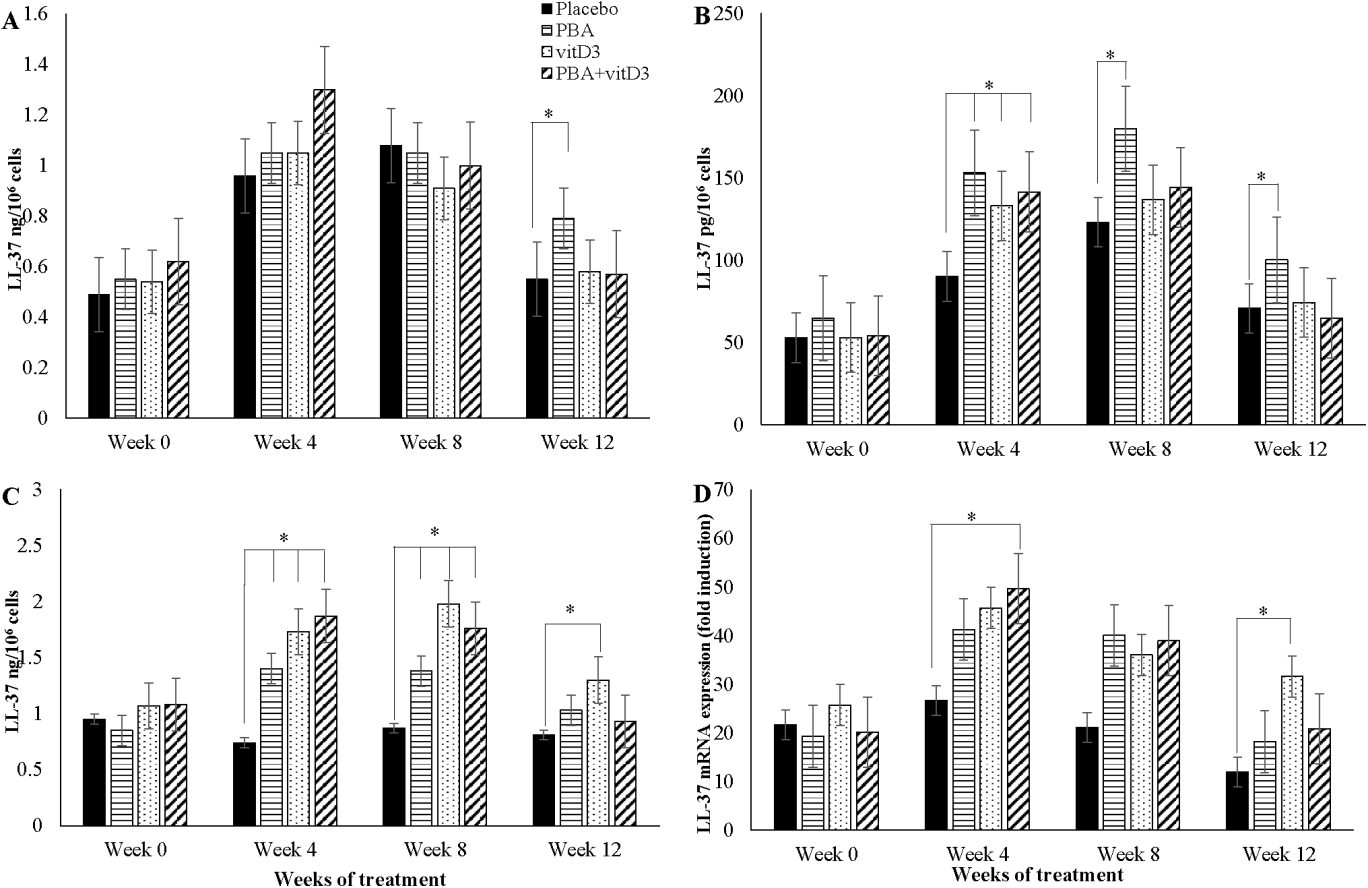
Concentration of antimicrobial peptide LL-37 at baseline, week 4, 8 and 12 after initiation of treatment in TB patients in the four intervention arms: (A) in monocyte-derived-macrophages (MDM); (B) in non-adherent lymphocytes; (C) in peripheral blood mononuclear cells (PBMC); (D) relative expression of LL-37 mRNA in MDM.

A significant increase in LL-37 peptide concentrations in MDM was found in the PBA-group compared to placebo at week 12 (*p* = 0.034) (**Fig 5A**). In addition, LL-37 peptide concentrations in lymphocytes from the PBA-, vitD_3_- and PBA+vitD_3_-groups were significantly higher at week 4, 8 and 12 compared to the placebo-group (*p* = 0.009, *p* = 0.053 and *p* = 0.022, respectively) (**Fig 5B**). In PBMC, LL-37 peptide levels increased significantly in the three intervention arms compared to placebo at week 4 (*p*<0.003 for all groups) and week 8 (*p*<0.030 for all groups), while increased LL-37 peptide levels at week 12 was found in the vitD_3_-group only compared to placebo, *p* = 0.030) (**Fig 5C**).

Concentration of LL-37 mRNA in MDM increased in the PBA+vitD_3_-group (*p* = 0.036) at week-4, in the PBA-group at week 8 (*p* = 0.057) and in the vitD_3_-group at week 12 (*p* = 0.003) compared to placebo (**Fig 5D**).

### Effects of adjunct therapy on effector function of macrophages

**Fig 6:**
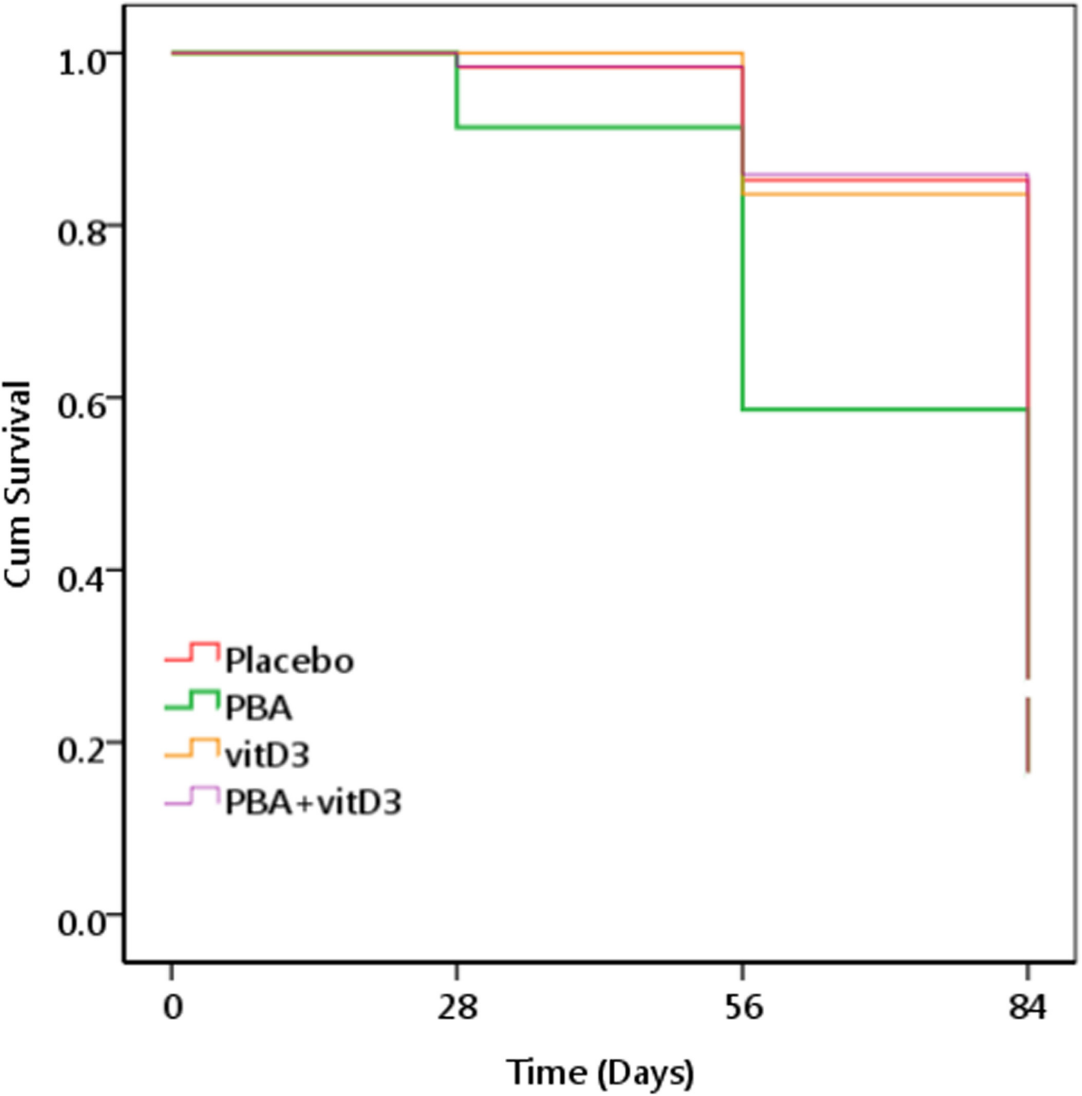
Kaplan Meier survival graph for monocyte-derived-macrophage (MDM)-mediated killing of *Mycobacterium tuberculosis (Mtb)*. Data are expressed as viability of *Mtb* in ‘relative CFU (colony forming unit) counts’. A ‘relative CFU count’ was calculated by normalizing the data in each time point with the inoculated *Mtb* CFU. A cut-off of 0.1 was considered as zero. PBA-group exhibited significantly earlier decline in intracellular *Mtb* CFU counts after MDM-mediated killing compared to the placebo-group.

Complete sets of macrophages were obtained from 244 TB patients at week 0, 4, 8 and 12. Within each group, the capacity of MDM to kill intra-cellular *Mtb* increased with time after initiation of anti-TB therapy as indicated by decreased viable *Mtb* count over time. We compared the survival of the intracellular *Mtb* in MDM according to intervention groups by plotting the Kaplan-Meier graph. The graph displays cumulative survival function of intracellular *Mtb* on a linear scale by intervention. In the log rank analysis, time to *Mtb* counts becoming zero was compared between the placebo and the intervention groups. The PBA-group demonstrated significantly earlier decline in viable *Mtb* cfu compared to the placebo-group (95% CI of time (days) to zero count, 65.3–74.7 vs. 76.5–82.3 respectively, log rank 11.38, *p* = 0.01) (**Fig 6**).

### Exploratory analyses

**Fig 7:**
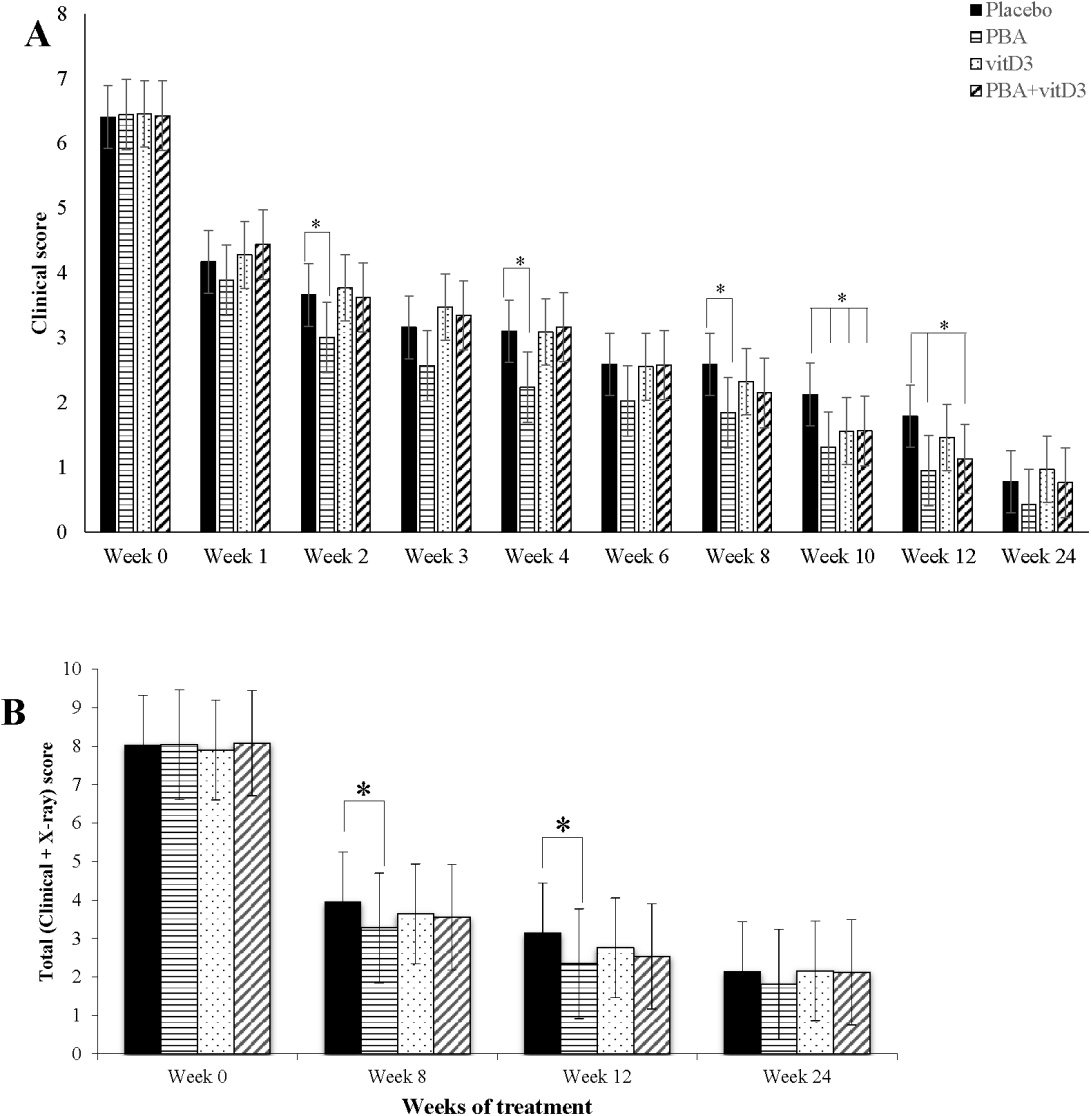
Mean TB score in TB patients in the four intervention arms during the study period. Standard deviation is shown as vertical bar. Comparisons of intervention arms are made with the placebo arm with statistically significant differences being shown in asterisks. The PBA-group demonstrated significantly lower TB scores than the placebo group at week 2, 4, 8, 10 and 12. At week 10 all three intervention groups showed lower scores than the placebo group. Multivariate regression analysis was utilized for comparison of mean effect of clinical scores in the different intervention groups.

The composite TB score (**S1 Table**) was calculated for patients at week 0, 1, 2, 3, 4, 6, 8, 10, 12 and 24 that excluded the chest x-ray score since x-ray was performed at fewer occasions. As age, sex, history of contact and BCG status had interaction effects on TB scores, we adjusted the clinical scores with these covariates using ANCOVA. The PBA-group showed a marked decline in TB score already at week 2, 4 and 8 compared to the placebo-group (*p* = 0.032, *p* = 0.006 and *p* = 0.026 respectively) (**Fig 7A**). However, at week 10 all three groups exhibited significantly lower score than the placebo-group (PBA, vitD_3_ or PBA+vitD_3_ vs. placebo (*p* = 0.003, *p* = 0.042 and *p* = 0.036 respectively). At week 12, TB score remained significantly lower in the PBA and PBA+vitD_3_ (*p* = 0.001 and *p* = 0.009) group compared to the placebo (**Fig 7A**).

At week 0, 8, 12 and 24, the composite TB score also included chest x-ray data. The TB score data were analyzed by ANCOVA after adjusting for age and sex. The complete data set for longitudinal analysis of chest x-ray was available for 211 out of the total of 219 TB patients. Reasons for missing results included chest x-ray plates that were insufficient or unavailable to the study physician. Here, the PBA-group showed significantly lower TB score compared to the placebo-group at week-8 (*p* = 0.042) and week-12 (*p* = 0.003) (**Fig 7B**).

Patients were stratified into vitD_3_-supplemented (vitD_3_ and PBA+vitD_3_) and non-vitD_3_ supplemented groups (PBA and placebo). The proportions of patients being culture-negative at week 4 and week 8 were higher in the vitD_3_-supplemented-group compared to non-vitD_3_-group (week 4, 66%; 84/127 vs. 44%; 54/122; p = 0.001) (week 8, 96.9%; 123/127 vs. 90.2%; 110/122; *p* = 0.031) respectively. Moreover in the vitD_3_-supplemented-group, the odds of sputum culture being negative at week 4 was 2.49 times (95% CI, 1.48–4.17, *p* = 0.001) and at week 8, 3.37 times (95% CI, 1.05–10.8, *p* = 0.041) higher compared to non-vitD_3_-group respectively. However, when clinical endpoints were considered, the odds of recovery from cough at week 2 was 2.42 (95% CI, 1.05–5.58) times lower in the vitD_3_-supplemented-group compared to the non-vitD_3_-group (p = 0.038).

## Discussion

In this clinical trial, we show positive effects of a novel ‘host-directed therapy’ by inducing the production of antimicrobial peptides (AMPs). Our hypothesis was that oral adjunct therapy with PBA alone or in combination with vitD_3_ to TB patients would increase the expression of the AMP LL-37, a marker for multiple AMPs, in alveolar macrophages and pulmonary epithelium and eventually accelerate elimination of *Mtb* bacilli from the respiratory tract. Adjunct therapy with vitD_3_ alone or in combination with PBA showed increased sputum culture conversion at week 4 and vitD_3_ alone at week 8 compared to the placebo group. Importantly, treatment with PBA, vitD_3_ or the combination also resulted in reduced clinical symptoms. In line with an improvement of the primary outcomes, treatment with PBA and vitD_3_ also resulted in a synergistic increased expression of LL-37 in immune cells that was paralleled with an enhanced intracellular killing of *Mtb* in macrophages *ex vivo* (**Fig 6**).

In similar clinical trials, the primary endpoint is usually culture conversion assessed at several time points after initiation of TB therapy. Although there are no published data on sputum culture conversion in Bangladesh, the smear conversion rate of Bangladeshi TB patients is estimated as fairly good ranging from 13 to 22 days (K Zaman, personal communication). Martineau *et al* have demonstrated that the median time to sputum culture conversion was 36 days in TB patients [17]. Accordingly, week 4 was selected as the readout time point for culture conversion in this study. Indeed, significant changes in the other outcome variables were also noted already at week 4 including reduction of TB scores, significantly elevated concentration of plasma 25(OH)D_3_, LL-37 mRNA, LL-37 peptide in PBMC and lymphocytes in the different intervention groups compared to the placebo. Significant changes in the same variables were also evident at week 8.

A significant effect of adjunctive therapy with PBA could be demonstrated using a composite TB score including resolution of symptoms [30, 41]. PBA alone was able to reduce clinical TB symptoms as early as the 2^nd^ week of treatment; the effect persisted until week 12 (**Fig 7**). Adding chest x-ray to the TB score further supported a beneficial effect of adjunct PBA treatment from week 8 to 12 [33]. The resolution of clinical symptoms was also reflected by reduced neutrophil counts in the PBA and PBA+vitD_3_ treatment groups. In this study, we did not find a decrease in the ratio of lymphocyte and monocyte as shown by Martineaue et al [17], however, monocyte counts alone declined significantly after adjunct therapy with PBA+vitD_3_ which is also suggestive of resolution of inflammation. Importantly, our significant finding on the TB scores should be interpreted with caution since the decline in TB scores was also significant in the placebo group. It is difficult to show an adjunctive effect of immunotherapy with PBA and vitD_3_ on top of the highly effective standard DOTS therapy. However, as our aim was to decrease the time of standard therapy and to reduce side effects, the biological effects of PBA and vitD_3_ may still be clinically relevant.

The role of 1,25(OH)_2_D_3_ as an inducer of LL-37 expression has previously been well-established [11]. Our *in vitro* studies have shown that PBA can induce the level of LL-37 mRNA and the production of the LL-37 peptide in both lung epithelial cells [24] and human macrophages [42]. Importantly, a synergistic effect on LL-37 expression was observed when 1,25(OH)_2_D_3_ was combined with PBA [24] as seen *ex vivo* in the current study. A recent *in vitro* study have been shown that, PBA together with vitamin D enhance the antimicrobial and anti-inflammatory effects against *Mtb* infection in macrophages [43]. We have also provided evidence that oral supplementation with PBA and vitD_3_ can induce LL-37 in MDM and lymphocytes in healthy volunteers that was linked to an improved killing of intracellular *Mtb* by infected macrophages *ex vivo* [26]. Here we show that the PBA+vitD_3_ and the vitD_3_-groups exhibited a more rapid sputum culture conversion detected already at week 4 compared to the placebo-group. Furthermore, enhanced bactericidal capacity of MDM was significant in the PBA group. The MDM was cultured from peripheral mononuclear cells of TB patients which might behave differently compared to tissue-resident macrophages, since circulating monocytes do not always end up replenishing tissue macrophage populations [44]. Our findings may indicate that there was a synergistic effect between PBA and vitD_3_ in elimination of bacteria from the lungs *in vivo* in the early phase of the treatment, where resident macrophages and additional immune cells have a major role.

Altogether, the findings of reduced TB scores, increased LL-37 concentration in MDM and increased MDM-mediated killing of *Mtb* in the PBA-group, suggest an important role of AMPs, such as LL-37, in the recovery from TB disease. Remission of clinical TB symptoms is probably through resolution of inflammatory responses as well as mycobacterial clearance from the body. Besides antimicrobial functions, LL-37 exhibits immune-modulatory activities [45] including both pro- and anti-inflammatory functions. For example, local effects of LL-37 include influences on cytokine/chemokine production, macrophage development and differentiation toward an inflammatory M1 phenotype [46–48] and enhanced phagocytosis by macrophages [49]. In addition, LL-37 can reduce pro-inflammatory cytokine production by down-regulation of signaling through TLR4 via binding to LPS and/or interrupt function of the TLR4 receptor complex in macrophages and dendritic cells [50–53]. Furthermore, LL-37 has been linked to receptor-mediated autophagy [42].

Our study confirms that there were no safety concerns regarding the daily oral doses of vitD_3_ and PBA that were given to the study patients. It is important to note that 91% of the Bangladeshi TB patients demonstrated low vitD_3_ status and 26–48% patients demonstrated hypocalcaemia at baseline. Treatment with vitD_3_ increased plasma levels of 25(OH)D_3_ in both vitD_3_ and PBA+vitD_3_ arms. However, there were no cases of hypercalcaemia, although hypocalcaemia persisted in patients who had low calcium at baseline. We found that the 2-month dose of vitD_3_ used in the trial enabled almost 100% patients to attain sufficient vitamin D status (>50 nmol/L). PBA is an FDA approved drug for urea cycle disorders [23] and a dose up to 10 g/day can be safely used for this purpose, which is about 10 fold higher compared to the dose used in the present trial (1g/day). There were no adverse events specifically linked to the PBA or vitD_3_ intervention, the majority of the adverse events was very likely due to standard anti-TB therapy.

The limitations of this study were that negative *Mtb* culture results were not examined for non-tuberculosis mycobacteria, although these patients received DOTS. The use of antibiotics (penicillin and streptomycin) in cell culture media for preventing contamination may have some effect on intracellular growth of *Mtb* in MDM. In line with other reports using vitD_3_ adjunct therapy in the treatment of TB, we also showed that the vitD_3_ intervention did not have an impact on time to sputum smear conversion [17, 54–56]. Importantly, in contrast to our findings, none of these clinical trials showed any impact on sputum culture conversion. Only, Martineau *et al* showed significantly hastened sputum culture conversion in a sub-group of patients with polymorphism in TaqI vitamin D receptor genotype with improved responsiveness to supplementation [17]. The strengths of our study are the convenience of dosing regimen of adjunctive therapy that matched with 2 months’ duration of 4-FDC in the standard treatment regimen of DOTS; the daily dose of vitD_3_ (5000 IU) for 2 months (total dose 300,000 IU) significantly increased the plasma 25(OH)D_3_ levels in TB patients that sustained for 6 months. In most TB trials, a high dose of vitamin D eg. up to 100,000 IU has typically been administrated at 3–4 occasions during standard anti-TB treatment, which has not provided any clear evidence of adjunctive treatment efficacy [54, 56, 57]. Importantly, the dose and/or dosing interval required for antimycobacterial therapy is not known and thus it may be insufficient to give such bolus doses. It has been proposed that daily dosing could be more beneficial than bolus dosing in the treatment of respiratory diseases [58, 59]. A dose of 150,000 IU of vitD_3_ given to Indonesian patients did not result in beneficial effects on TB outcomes [54]. Two intramuscular doses of 600,000 IU of vitD_3_ in Pakistani patients resulted in greater weight gain and rapid radiographic clearance of the disease in the intervention group. However, concentration of vitD_3_ was not determined after supplementation in either study, thus it was not clear if the clinical improvement was associated with elevated 25(OH)D_3_ levels [55]. In a trial in Guinea-Bissau, a total dose of 300,000 IU did not result in higher serum 25(OH)D_3_ in the intervention group compared to the controls [56]. Also opposed to our study, the TB patients in two of the other trials were not vitamin D insufficient or deficient at baseline (60 nmol/L), which is probably an important prerequisite to observe a positive clinical effect of vitD_3_ supplementation [54, 55]. We assumed that vitamin D has to be administered in a concentrated dose (5000 IU/day) at much earlier and more frequent time points (i.e. daily for 2 months) in order to have an optimal antimycobacterial effect and to significantly shorten the duration of standard anti-TB chemotherapy. Moreover, PBA was added as an additional adjunct therapeutic compound for enhanced impact.

The therapeutic potential of LL-37 in various infections has been demonstrated by *in vitro* experiments, but efficient use of elicitors of AMPs in clinical studies is rare. There is a great interest in the use of nutritional agents or repurposed drugs that can be used as adjunct therapy to treatment of infectious diseases. Tailoring the use of such agents as host-directed or pathogen-directed therapies that can restore clinically relevant immune responses can be a novel approach [60]. Altogether, the findings of this clinical trial are encouraging; there are indications that this type of host-directed therapy can be exploited to fight respiratory infections by enhancing innate immunity, antimicrobial activity and modulating immune responses. Further studies are needed to understand the role of PBA and vitD_3_ in reducing inflammatory responses or restoring appropriate immune responses in the treatment of MDR-TB, extra-pulmonary TB as well as TB and associated co-morbidities including diabetes or HIV.

## Supporting Information

S1 CONSORT Checklist CONSORT Checklist. (DOCX)

S1 Protocol Study Protocol. (PDF)

S1 Table Clinical parameters used for clinical scores. (DOCX)

S2 Table Differences between baseline characteristics when randomized patients were compared to potentially eligible patients who declined or had to be excluded from the study. (DOCX)

S3 Table Vitamin D (25-hydroxyvitamin D_3_) status of TB patients at baseline and at 8 and 24 weeks after initiation of adjunctive therapy. (DOCX)

S1 Text Consent. Sample informed consent form. (PDF)

S2 Text Report. Sample case report form. (PDF)

## Abbreviations

1,25(OH)_2_D_3_: 1,25-dihydroxyvitamin D_3_
25(OH)D_3_: 25-Hydroxyvitamin D_3_
4-FDC: 4 drug fixed-dose-combination
AE: Adverse events
BCG: Bacillus Calmette–Guérin
CFU: colony forming units
CRP: C-reactive protein
DEQAS: vitamin D External Quality Assessment Scheme
DOTS: Directly Observed Treatment, Short-Course
DST: Drug susceptibility tests
ECF: extracellular fluid
ESR: erythrocyte sedimentation rate
icddr,b: International Centre for Diarrheal Disease Research, Bangladesh
ICF: intracellular fluid
L-J: Lowenstein-Jensen
MDM: monocyte-derived macrophages
MDR-TB: multidrug-resistant tuberculosis
*Mtb*: *Mycobacterium tuberculosis*
MOI: multiplicity of infection
NIDCH: National Institute of the Diseases of the Chest and Hospital
PBA: 4-phenylbutyrate
PBMC: peripheral blood mononuclear cells
SAE: Serious adverse events
TB: tuberculosis
vitD_3_: vitamin D_3_
