# Supplementary material for: Significant Effects of Oral Phenylbutyrate and Vitamin D3 Adjunctive Therapy in Pulmonary Tuberculosis: A Randomized Controlled Trial
Source: PLoS One. 2015 Sep 22;10(9):e0138340. doi: 10.1371/journal.pone.0138340 (PMC4578887; doi:10.1371/journal.pone.0138340)
Supplement: S1 Protocol — (PDF) [file pone.0138340.s002.pdf]

icddr,b KNOWLEDGE FOR GLOBAL LIFESAVING SOLUTIONS	**RRC APPLICATION FORM**

**RESEARCH PROTOCOL NUMBER: PR-09068**	**FOR OFFICE USE ONLY**			
	RRC Approval:	☐ Yes /	☐ No	Date:
	ERC Approval:	☐ Yes /	☐ No	Date:09 Dec2009
	AEEC Approval:	☐ Yes /	☐ No	Date:
	External IRB Approval: Name of IRB:	☐ Yes /	☐ No	Date:

Protocol Title: Clinical trial of oral phenylbutyrate and vitamin D adjunctive therapy in pulmonary tuberculosis in Bangladesh: a pilot study

Short title (in 50 characters including space): Clinical trial of phenylbutyrate and vitamin D in TB

Theme: (Check all that apply)

- ☐ Nutrition
- ☒ Emerging and Re-emerging Infectious Diseases
- ☐ Population Dynamics
- ☐ Reproductive Health
- ☐ Vaccine Evaluation
- ☐ HIV/AIDS
- ☐ Environmental Health
- ☐ Health Services
- ☐ Child Health
- ☐ Clinical Case Management
- ☐ Social and Behavioral Sciences
- ☐ Gender
- ☐ Human Rights
- ☐ Others (please specify____________________)

Key words: tuberculosis, phenylbutyrate, vitamin D, cathelicidin, antimicrobial peptide

Relevance of the Protocol:

Tuberculosis (TB) caused by *Mycobacterium tuberculosis* (MTB) is a major public health problem worldwide which is responsible for over 3 million deaths annually. Recent analysis of global burden of TB revealed that Bangladesh ranks as the 6th highest among 212 countries. The global prevalence of TB is increasing due to the spread of multidrug-resistant strains of MTB with HIV pandemic being a strong factor in the resurgence of TB. Concomitant occurrence of TB and HIV presents a lethal combination. The recent emergence of extensively drug resistant (XDR) tuberculosis in 50 countries from all regions of the world, including industrialized nations, poses a grave global threat to human health. Against the backdrop of heightened threat posed by this newly emerged often untreatable and fatal tuberculosis and to combat the global problem successfully, research into development of new classes of anti-TB drugs and alternative treatment strategies are urgently required. By assessing the efficacy of adjunctive therapy with vitamin D and Phenylbutyrate, this proposal may help to develop novel alternative methods in combating tuberculosis by pharmacologically up-regulating endogenous antibiotic peptides. Results from this study may guide and provide support to the policy makers to identify effective strategies to treat MDR as well as XDR TB.

Centre's Priority (as per Strategic Plan, to be imported from the attached Separate Word Sheet): # 17. Define the epidemiology and burden of selected infectious diseases and identify effective strategies for prevention and control. These include tuberculosis, diarrhoea, ALRI (pneumonia), typhoid fever, dengue, malaria, kala azar, and drug-resistant infections.

Programmes:

- ☐ Child Health Programme
- ☐ Nutrition Programme
- ☒ Programme on Infectious Diseases & Vaccine Science
- ☐ Poverty and Health Programme
- ☐ Health and Family Planning Systems Programme
- ☐ Population Programme
- ☐ Reproductive Health Programme
- ☐ HIV/AIDS Programme
- ☐ Gender, Hunman Rights and Health Programme
- ☐ Others (please specify____________________)

**Principal Investigator (Should be a Centre's staff)**<br>Rubhana Raqib (F)<br>**Address (including e-mail address):**<br>rubhana@icddrb.org	**DIVISION:**<br>☐ CSD ☒ LSD<br>☐ HSID ☐ PHSD

Co-Principal Investigator(s): Internal

Co-Principal Investigator(s): External: Swedish
(Please provide full official address including e-mail address and Gender)
Birgitta Agerberth (F), Karolinksa Institutet

Co-Investigator(s): Internal:
Zeaur Rahim, Dinesh Mondal

Co-Investigator(s): External (Please provide full official address including e-mail address and Gender)
SM Mostafa Kamal (M); Asif Mustaba Mahmud (M); Jan Andersson (M); Gudmundur Gudmundsson (M)

Student Investigator(s): Internal (Centre's staff):

Student Investigator(s): External: (Please provide full address of educational institution and Gender)

Collaborating Institute(s): Please Provide full address

Institution # 1

Country	Sweden
Contact person	Professor Birgitta Agerberth, PhD
Department<br>(including Division, Centre, Unit)	Department of Medical Biochemistry and Biophysics (MBB)<br>Chemistry I, Scheeles väg 2, A3, Plan5
Institution<br>(with official address)	Karolinska Institutet<br>S-171 77 Stockholm, SWEDEN

Institution # 2

Country	Sweden
Contact person	Professor Jan Andersson, MD, PhD
Department<br>(including Division, Centre, Unit)	Division of Infectious Diseases, Department of Medicine,<br>I-63, Plan 6
Institution<br>(with official address)	Karolinska University Hospital, S-14186 Huddinge

Institution # 3

Country	Bangladesh
Contact person	Dr SM Mostafa Kamal, MD, MPhil
Department<br>(including Division, Centre, Unit)	National Tuberculosis Reference Laboratory
Institution<br>(with official address)	National Institute of Diseases of Chest and Hospital (NIDCH) ,<br>Mohakhali, Dhaka-1212
Directorate<br>(in case of GoB i.e. DGHS)	DGHS
Ministry (in case of GoB)	MOHSFW

Institution # 3

Country	Bangladesh
Contact person	Dr. Asif Mustaba Mahmud, MD, PhD
Department<br>(including Division, Centre, Unit)	Respiratory Medicine
Institution<br>(with official address)	National Institute of Diseases of Chest and Hospital (NIDCH) ,<br>Mohakhali, Dhaka-1212
Directorate<br>(in case of GoB i.e. DGHS)	DGHS
Ministry (in case of GoB)	MOHSFW

Institution 3

Country	Bangladesh
Contact person	Assistant Prof. Dr. Md Naimul Hoque, MBBS, DTCD, FCCP
Department<br>(including Division, Centre, Unit)	Respiratory Medicine
Institution<br>(with official address)	National Institute of Diseases of Chest and Hospital (NIDCH) ,<br>Mohakhali, Dhaka-1212
Directorate<br>(in case of GoB i.e. DGHS)	DGHS
Ministry (in case of GoB)	MOHSFW

Institution #4

Country	Iceland
Contact person	Professor Gudmundur Gudmundsson, PhD
Department<br>(including Division, Centre, Unit)	Biology Institute
Institution (with official address)	University of Iceland,<br>Sturlugata 7, 101 Reykjavik

Population: Inclusion of special groups (Check all that apply):

Sex
- ☒ Male
- ☒ Female

Age
- ☐ 0 – 4 years
- ☐ 5 – 10 years
- ☐ 11 – 17 years
- ☒ 18 – 64 years
- ☐ 65 +

- ☐ Pregnant Women
- ☐ Fetuses
- ☐ Prisoners
- ☐ Destitutes
- ☐ Service Providers
- ☐ Cognitively Impaired
- ☐ CSW
- ☐ Others (specify)
- ☐ Animal

NOTE It is the policy of the Centre to include men, women, and children in all research projects involving human subjects unless a clear and compelling rationale and justification (e.g. gender specific or inappropriate with respect to the purpose of the research) is there. Justification should be provided in the `Sample Size' section of the protocol in case inclusiveness of study participants is not proposed in the study.

Project/study Site (Check all the apply):

- ☐ Dhaka Hospital
- ☐ Matlab Hospital
- ☐ Matlab DSS Area
- ☐ Matlab non-DSS Area
- ☐ Mirzapur
- ☐ Dhaka Community
- ☐ Chakaria
- ☐ Abhoynagar
- ☐ Mirsarai
- ☐ Patyia
- ☒ Other areas in Bangladesh: NIDCH, Mohakhali
- ☐ Outside Bangladesh
  Name of Country:
- ☐ Multi Centre Trial

(Name other countries involved):

Type of Study (Check all that apply):

- ☐ Case Control Study
- ☐ Community-based Trial/Intervention
- ☐ Program Project (Umbrella)
- ☐ Secondary Data Analysis
- ☒ Clinical Trial (Hospital/Clinic)
- ☐ Family Follow-up Study
- ☐ Cross Sectional Survey
- ☐ Longitudinal Study (cohort or follow-up)
- ☐ Record Review
- ☐ Prophylactic Trial
- ☐ Surveillance/Monitoring
- ☐ Others:

NOTE: Does the study meet the definition of clinical studies/trials given by the International Committee of Medical Journal Editors (ICMJE)? Yes ☒ No ☐

Please note that the ICMJE defined clinical trial as "*Any research project that prospectively assigns human subjects to intervention and comparison groups to study the cause-and-effect relationship between a medical intervention and a health outcome*".

If YES, after approval of the ERC, the PI should complete and send the relevant form to provide required information about the research protocol to the Committee Coordination Secretariat for registration of the study into websites, preferably at the https://register.clinicaltrials.gov/. It may please be noted that the PI would require to provide subsequent updates of the research protocol for updating protocol information in the website.

Targeted Population (Check all that apply):

- ☒ No ethnic selection (Bangladeshi)
- ☐ Bangalee
- ☐ Tribal group
- ☐ Expatriates
- ☐ Immigrants
- ☐ Refugee

Consent Process (Check all that apply):

- ☒ Written
- ☐ Oral
- ☐ None
- ☒ Bengali Language
- ☒ English Language

Proposed Sample Size:

Sub-group (Name of subgroup (e.g. Men, Women) and Number

Name	Number	Name	Number
Healthy subjects	9		
(1) Patients receiving placebo	72	(3) Patients receiving sodium phenyl butyrate	72
(2) Patients receiving vit D	72	(4) Patients receiving vit D plus sodium phenyl butyrate	72

Total sample size: 297

a) Will the specimen be stored for future use? Yes ☑ No ☐

b) If yes, how long the specimens be preserved? 8 years.

c) Will consent be obtained from study participants for the specimen be stored for future, for unrelated use without further taking consent? Yes ☑ No ☐ NA ☐

d) What types of tests will be carried out with the preserved samples? New markers of innate immunity

e) Will the samples be shipped to other country(ies)? Yes ☑ No ☐ NA ☐

f) If yes, name of institution(s) and country(ies): Karolinksa Institutet, Sweden

g) Will the surplus/unused specimen be returned to the Centre? Yes ☑ No ☐ NA ☐

h) Who will be the custodian of the specimen at the Centre and when shipped outside of the country(ies)?: PI at ICDDR,B (Rubhana Raqib)

i) Who will be the owner(s) of the samples? : PI at ICDDR,B

j) Has a MoU been made for the protocol covering the specimen collection, storage, use and ownership? Yes ☐ No ☑ NA ☐

k) If yes, please attach a copy.

Determination of Risk: Does the Research Involve (Check all that apply**):**

- ☐ Human exposure to radioactive agents?
- ☐ Fetal tissue or abortus?
- ☐ Investigational new device? (specify:)
- ☐ Existing data available from Co-investigator
- ☐ Human exposure to infectious agents?
- ☐ Investigational new drug
- ☐ Existing data available via public archives/sources
- ☒ Pathological or diagnostic clinical specimen only
- ☐ Observation of public behaviour
- ☐ New treatment regime

Yes	No	
☒	☐	Is the information recorded in such a manner that study participants can be identified from information provided directly or through identifiers linked to the study participants?
☐	☒	Does the research deal with sensitive aspects of the study participants' behaviour; sexual behaviour, alcohol use or illegal conduct such as drug use?

Could the information recorded about the individual if it became known outside of the research:

Yes	No	
☐	☒	Place the study participants at risk of criminal or civil liability?
☐	☒	Damage the study participants' financial standing, reputation or employability, social rejection, lead to stigma, divorce etc.?

Do you consider this research (Check one):

☐ Greater than minimal risk ☒ No more than minimal risk

☐ Only part of the diagnostic test

Minimal Risk is the risk when the probability and magnitude of the anticipated harm or discomfort in participating in the proposed research are not greater in and of themselves than those ordinarily encountered in daily life or during the performance of routine physical, psychological examinations or tests, e.g. the risk of drawing a small amount of blood from a healthy individual for research purposes is no greater than the risk of doing so as a part of routine physical examination.

Yes/ No

☒ ☐ Is the proposal funded?

If yes, sponsor Name: (1) Sida/SAREC

Yes/No/NA (if the proposal is already funded, mark NA)

☐ ☒ Is the proposal being submitted for funding?

If yes, name of funding agency: (1)
(2)

Do any of the participating investigators and/or member(s) of their immediate families have an equity relationship (e.g. stockholder) with the sponsor of the project or manufacturer and/or owner of the test product or device to be studied or serve as a consultant to any of the above?

IF YES, a written statement of disclosure to be submitted to the Centre's Executive Director.

Dates of Proposed Period of Support

(Day, Month, Year - DD/MM/YY)

Beginning Date : As soon as possible

End Date : Two years from starting

Cost Required for the Budget Period ($)

**Years**	**Direct Cost**	**Indirect Cost**	**Total Cost**
**Year-1**			
**Year-2**			
**Year-3**			0
**Year-4**			0
**Year-5**			0
**Total**			

Certification by the Principal Investigator

I certify that the statements herein are true, complete and accurate to the best of my knowledge. I am aware that any false, fictitious, or fraudulent statements or claims may subject me to criminal, civil, or administrative penalties. I agree to accept the responsibility for the scientific conduct of the project and to provide the required progress reports including updating protocol information in the SUCHONA (Form # 2) if a grant is awarded as a result of this application.

**Signature of PI**	**Date**

Approval of the Project by the Division Director of the Applicant

The above-mentioned project has been discussed and reviewed at the Division level as well by the external reviewers. The protocol has been revised according to the reviewers' comments and is approved.

Hubert Endtz		
Name of the Division Director	Signature	Date of Approval

Table of Contents

RRC APPLICATION FORM..1
Project Summary...8
Description of the Research Project...8
Hypothesis to be Tested:...9
Specific Aims:... 9
Background of the Project including Preliminary Observations...9
Research Design and Methods..................................... 11
Sample Size Calculation and Outcome Variable(s)...15
Facilities Available...15
Data Safety Monitoring Plan (DSMP) 16
Data Analysis...16
Ethical Assurance for Protection of Human Rights 16
Use of Animals 17
Literature Cited...17
Dissemination and Use of Findings..18
Collaborative Arrangements...19
Itemized specific tasks and time frame...19
Budget Justifications.............. 19
Budget 21
Other Support 22
Appendix 1: Case Report Form
Appendix II: Data from Manuscript
Appendix III: Material safety sheet of Phenylbutyrate
Appendix IV: Basis of Allometry calculations

☒ Check here if appendix is included

Project Summary:

Principal Investigator: Rubhana Raqib

Research Protocol Title: Clinical trial of oral phenylbutyrate and vitamin D adjunctive therapy in pulmonary tuberculosis in Bangladesh: a pilot study

Total Budget US$: Beginning Date : As soon as possible Ending Date: Two years from starting

Tuberculosis (TB) caused by *Mycobacterium tuberculosis* (MTB) is a major public health problem worldwide which is responsible for over 3 million deaths annually. Recent analysis of global burden of TB revealed that Bangladesh ranks as the 6th highest among 212 countries. The prevalence of TB is increasing due to the spread of multidrug-resistant strains of MTB with HIV pandemic being a strong factor in the resurgence of TB. Concomitant occurrence of TB and HIV presents a lethal combination. The recent emergence of extensively drug resistant (XDR) tuberculosis in 50 countries from all regions of the world, including industrialized nations, poses a grave global threat to human health. Against the backdrop of heightened threat posed by the newly emerged often untreatable and fatal tuberculosis and to combat the global problem successfully, research into development of new classes of anti-TB drugs and alternative treatment strategies are urgently required.

Earlier epidemiological studies suggested a link between vitamin D deficiency and TB. It is now known that Vitamin D exerts its effects via the Vitamin D Receptor (VDR) present in activated macrophages and induces expression and release of the cathelicidin, LL-37, a human antimicrobial peptide involved in killing of MTB. Our group has earlier shown that treatment with sodium butyrate, enhances cathelicidin expression in intestinal cell line and *in vivo* in colonic epithelium of a rabbit model with rapid clinical recovery and concomitant decline in bacterial load in stool. We have further shown that sodium phenylbutyrate (PBA) an analogue of butyric acid and a registered drug used for the treatment of urea cycle disease exhibits the same capacity as butyrate to induce the production of LL-37 in bronchial epithelial cell line VA10. A synergistic effect between PBA and active vitamin D (1,25$(OH)_2$D3) in inducing LL-37 expression in these cells was also noted. Interestingly, PBA and active vitamin D separately and in combination could enhance macrophage mediated killing of MTB.

We thus aimed to investigate whether treatment of newly diagnosed pulmonary TB patients for 2 months with adjunctive PBA and vitamin D (Cholecalciferol) along with standard anti-TB therapy (i) can improve response to standard short course TB therapy towards a rapid recovery; (ii) can induce expression of LL-37 in macrophages; (iii) can enhance killing capacity of macrophages isolated from TB patients infected *in vitro* with MTB; and (iv) does not evoke any adverse effects. Before starting the clinical trial, 3 different doses of PBA (250 mg, 500 mg and 1000 mg per dose, twice daily together with 5000 IU of vit D3 once daily) for 4 days will be tested in healthy individuals in 3 groups consisting of 3 participants per group. Blood will be collected on day-0, day-4 and day-8 to assess the concentration of LL-37 in macrophages; based on these results the dose to be tested in TB patients will decided.

KEY PERSONNEL (List names of all investigators including PI and their respective specialties)

Name	Professional Discipline/ Specialty	Role in the Project
1. Rubhana Raqib	Immunology/ Infectious diseases/Biochemistry	PI
2. Birgitta Agerberth	Medical Biochemistry	Co-PI
3. Jan Andersson	Infectious diseases/Immunology/Clinical trials	Co-investigator
4. Gudmundur Gudmundsson	Cell Biology	Co-investigator
5. Zeaur Rahim	Molecular Microbiology/Tuberculosis	Co-investigator
6. Dinesh Mondal	Tuberculosis/ Paediatrics	Co-investigator
7. SM Mostafa Kamal	Microbiology/Tuberculosis	Co-investigator
8. Asif Mustaba Mahmud	Respiratory Medicine /Tuberculosis	Co-investigator
9. Md Naimul Hoque	Respiratory Medicine /Tuberculosis	Co-investigator

Description of the Research Project

Please briefly list the Hypothesis to be tested and provide the scientific basis of the hypothesis, critically examining the observations leading to the formulation of the hypothesis.

Hypothesis to be Tested:

Adjunctive treatment (2 months) together with regular anti-TB therapy of newly diagnosed pulmonary TB patients with sodium phenylbutyrate and vitamin D:

1. improves response to standard short course TB therapy towards a rapid recovery (clinical, radiological, mycobacterial).
2. induces expression of LL-37 in macrophages (immunological).
3. enhances killing capacity of macrophages from TB patients infected *in vitro* with MTB (functional measures of treatment outcome).

Specific Aims:

Describe the specific aims of the proposed study. State the specific parameters, biological functions, rates, processes etc. that will be assessed by specific methods.

Objective 1: To determine the minimum oral dose of PBA required for induction of antimicrobial peptide in macrophages from healthy adults.

Objective 2

The second aim of this study is to determine whether adjunctive sodium phenylbutyrate and vitamin D treatment (2 months) of patients with newly diagnosed pulmonary TB :

1. Can improve response to standard short course TB therapy towards a rapid recovery (clinical, radiological, mycobacterial).
2. Can induce expression of LL-37 in macrophages (immunological).
3. Can enhance killing capacity of macrophages from TB patients infected *in vitro* with MTB (functional measures of treatment outcome).

Background of the Project including Preliminary Observations

Provide relevant background of the proposed study, and discuss the previous works on the research topic by citing specific references. Describe in a logical way how the present hypothesis is supported by the relevant background observations including any preliminary results that may be available. Provide scientific validity of the hypothesis on the basis of background information Critically analyze available knowledge in the field of the proposed study and discuss the questions and gaps in the knowledge that need to be fulfilled to achieve the proposed goals.. If there is no sufficient information on the subject, indicate the need to develop new knowledge. Also include

the significance and rationale of the proposed work by specifically discussing how these accomplishments will bring benefit to human health in relation to biomedical, social, and environmental perspectives.

Tuberculosis (TB) is a predominant public health problem worldwide and responsible for over 3 million deaths annually and is caused by *Mycobacterium tuberculosis* (MTB) [1]. About 2 billion persons are already infected world wide and 8-12 million new clinical cases occur each year [1, 2]. Recent analysis of global burden of TB revealed that Bangladesh ranks as the sixth highest among 212 countries. The prevalence of TB is increasing due to the spread of antibiotic-resistant strains of MTB and the deleterious consequences of co-infection with HIV [2]. Recent emergence of extended drug resistance (XDR) cases in South-Africa is associated with catastrophic consequences for many TB patients Thus, it is clear that advances in antituberculous therapies and alternative treatment strategies are urgently required both for TB patients and those at risk of developing the disease.

Endogenous antimicrobial peptides are essential effectors of the innate immune system [3] that are of great importance in host defence against bacteria [4, 5]. There are two major classes of these peptides in mammals: the defensins (reviewed in [6]) and cathelicidins [7]. The peptides are secreted from neutrophils and found in the bloodstream, and on epithelial mucosal surfaces of mammals, including lungs. The human antimicrobial peptide LL-37 is the only member of cathelicidins identified in humans and this active mature peptide is processed from its precursor hCAP-18 [7]. Hormonal induction of the CAMP gene encoding LL-37 was established when 1,25-dihydroxyvitamin D_3 was demonstrated as an inducer of LL-37 expression. A Vitamin D responsive element (VDRE) in the gene promoter was shown to bind the Vitamin D receptor (VDR) and to be necessary for the induction [8, 9]. Interestingly, it was recently shown that activation of Toll-like receptors (TLRs) in human macrophages upregulates the vitamin D receptor (VDR) and the vitamin D_1 hydroxylase genes, leading to the induction of LL-37 with subsequent killing of intracellular *Mycobacterium tuberculosis* [10]. It was also observed that low levels of 25-hydroxyvitamin D3 in sera of African-American individuals, which are more susceptible to tuberculosis, were less efficient to induce the expression of LL-37 [10]. This indicates a connection between low LL-37 levels and susceptibility to TB infection and that vitamin D plays a key role in the production of LL-37, which kills the TB bacteria [10]. Another study reported that UVB light can stimulate expression of LL-37 most likely through a chain of events that involves activation of Vitamin D [9]. Vitamin D exerts its effects via the VDR present in activated macrophages. The precursor $25(OH)D_3$ can be hydroxylated into its active form $1,25(OH)_2D_3$ in the macrophages. When the active form of Vitamin D binds to its receptor, the receptor-ligand complex is translocated into the nuclei and binds to the promoter of the *CAMP* (cathelicidin antimicrobial peptide) gene encoding LL-37 with subsequent expression and release of LL-37 peptide and killing of MTB [10]. This suggests a mechanism through which vitamin D stimulates immune defences against this pathogen.

Before the discovery of effective antimycobacterial drugs, Vitamin D therapy in the form of cod liver oil and exposure to sunlight (heliotherapy) were used to treat human tuberculosis [11], with striking effects in many patients [12]. The body produces the active or hormonal form of vitamin D when sunlight hits the skin. The skin pigment melanin more abundant in darker skin shields the body from the sun's rays, reducing damage from ultraviolet light as well as reducing vitamin D production. A study of the response of macrophages cultured in serum from African Americans showed that these subjects produced less LL-37 than cells cultured in serum from whites [10]. Adding a vitamin D precursor to the African-American serum culture increased LL-37 production. Thus, this study indicates that people of African descent though living in equator region are more susceptible to TB than Caucasians, with higher rates of infection and more severe cases once infected. This finding also suggests that a cheap dietary supplement can be rapidly distributed to the endemic areas which may be effective in reducing the frequency and severity of one of the world's most lethal diseases [13].

Epidemiological evidence suggests a link between vitamin D deficiency and TB. Several studies have shown lower levels of serum vitamin D in untreated TB patients compared to matched controls

[12]. On one hand, it is possible that a fall in serum vitamin D levels compromises cell mediated immunity and leads to the activation of latent TB or predisposing individuals to TB [14]. On the other hand, TB disease itself may cause lower levels [15]. It has also been suggested that anti-TB treatment with isoniazid or rifampicin may lower serum vitamin D levels [16]. There are no conclusive studies differentiating vitamin-D deficiency being a risk factor for TB and TB-induced nutritional deficiency causing low concentrations of vitamin D. Although according to a recent meta-analysis, it is more likely that low body vitamin D levels increase the risk of active tuberculosis [17]. Thus, there is a lack of prospective randomized controlled trials examining the effect of vitamin D in TB and the association of the induced levels of LL-37.

Previous research: We have observed a down-regulation of the cathelicidin LL-37 in dysenteric diarrhea and cholera [18] and in cells infected with *Neisseria gonorrhoeae* [19], indicating a subversive strategy of pathogenic bacteria in order to evade the host's first line of defence. These bacteria are able to turn off the expression of endogenous antimicrobial peptides, resulting in serious infections. We further demonstrated an induction of LL-37 with butyrate in colon epithelial cells *in vitro* [20]. We have also demonstrated the downregulation of the rabbit homologue to LL-37 (CAP-18) in a rabbit model of shigellosis [21]. As a proof-of-principle, by treatment with sodium butyrate, a by-product of dietary fibre digestion in the colon, we have further shown that the production of CAP-18 is restored with concomitant decline in bacterial load in the stool and rapid clinical recovery [21]. Since butyrate is a foul smelling compound, we have investigated non-smelling derivatives of butyrate. Phenylbutyrate (PBA) is one such analogue of butyric acid and we have shown that it exhibits the same capacity as butyrate to induce the production of LL-37 (Manuscript submitted)**.** PBA is a registered drug used primarily for the treatment of urea cycle disease [22]. We have demonstrated that the dose required for the antimicrobial effect appears to be much lower (see safety consideration, pg 13) than that indicated for the current treatment, signifying that safety is of minimal concern. Thus, PBA can be directly utilized for clinical studies without further safety trials.

Preliminary data: We have recently shown that PBA upregulates the expression of the *CAMP* gene encoding the cathelicidin LL-37 both on mRNA and protein levels in the human bronchial epithelial cell line VA10 (Manuscript in preparation). Interestingly, we also demonstrated a synergistic effect between PBA and the active form of vitamin D ($1,25(OH)_2D_3$) in inducing the expression of LL-37 in this cell line. Furthermore, we assessed the *in vitro* effects of PBA and $1,25(OH)_2D_3$, respectively, and in combination on macrophage mediated killing of MTB. The result shows a synergistic effect of PBA and $1,25(OH)_2D_3$ in the killing capacity of macrophages (Table 1).

Table 1: CFU count of MTB after overnight exposure of macrophages to different concentration of PBA and/or active Vitamin D [$1,25(OH)_2D_3$]

MOI	Substance	CFU count	Substance	CFU count
$1:10^2$<br>$1:10^3$<br>$1:10^4$	Media only	5<br>125<br>200	Media only	3<br>46<br>336
$1:10^2$<br>$1:10^3$<br>$1:10^4$	4mM PBA	5<br>110<br>150	20nM Vit-D<br>+ 4mM PBA	No colony<br>10<br>60
$1:10^2$<br>$1:10^3$<br>$1:10^4$	10mM PBA	No colony<br>35<br>170	20nM Vit-D<br>+10mM PBA	No colony<br>10<br>15
$1:10^2$	20mM PBA	No colony	20nM Vit-D	1

$1:10^3$<br>$1:10^4$		45<br>200	+ 20mM PBA	2<br>9
$1:10^2$<br>$1:10^3$<br>$1:10^4$	20 nM vit D	1<br>15<br>472	20 nM vitD<br>+ 40 mM PBA	1<br>No colony<br>8
$1:10^2$<br>$1:10^3$<br>$1:10^4$	80 nM vit D	3<br>16<br>166	20 nM vitD<br>+ 60 mM PBA	No colony<br>2<br>3

Research Design and Methods

Describe in detail the methods and procedures to be used in accomplishing the objectives and specific aims of the project. Discuss the alternative methods that are available and justify the use of the method proposed in the study. Justify the scientific validity of the methodological approach (biomedical, social, or environmental) as an investigation tool to achieve the specific aims. Discuss the limitations and difficulties of the proposed procedures and sufficiently justify the use of them. Discuss the ethical issues related to biomedical and social research for employing special procedures, such as invasive procedures in sick children, use of isotopes or any other hazardous materials, or social questionnaires relating to individual privacy. Point out safety procedures to be observed for protection of individuals during any situations or materials that may be injurious to human health. The methodology section should be sufficiently descriptive to allow the reviewers to make valid and unambiguous assessment of the project.

Phase 1:

A phase I dose escalation trial will be conducted in healthy subjects. Healthy participants (n=9, age 18-60 yrs) will be recruited from areas situated around ICDDR,B (or healthy attendants of patients coming to the Hospital). There will be 3 groups and in each group there will be three participants. The subjects will be followed for 8 days. Three doses will be given orally, 250 mg, 500 mg or 1000 mg, twice daily for 4 days together witha daily dose of vitamin D3 as given in Table 2. Blood will be collected on the day of recruitment (day-1), after 4 days of treatment (day-4) and 4 days after the last dose (day-8 from enrolment). Concentration of LL-37 will be determined in macrophages and based on the results, the minimum dose will be selected for the clinical trial in adult patients with TB.

Table 2. The dose given to each group and the specimen collected from healthy volunteers.

Healthy volunteers	Vitamin D	PBA	Specimen	Day-1	Day-4	Day-8
Group I	5000 IU/day	250 mgx2/day	Blood			
Group II	5000 IU/day	500 mgx2/day	Blood			
Group III	5000 IU/day	1000 mgx2/day	Blood			

Note. The dose will be given for 4 days.

Phase II:

Study subjects and area: Patients (age range 15-60 years) attending the outpatient of the National Institute of Diseases of Chest and Hospital (NIDCH) in Dhaka who are freshly diagnosed with smear positive pulmonary tuberculosis will be recruited. New sputum smear-positive pulmonary TB patients are Category I patients (Case Definitions according to World Health Organization; http://www.who.int/tb/publications/cds_tb_2003_313/en/).

Eligibility:

Inclusion Criteria:

- Adults, 18-60 years with sputum smear positive pulmonary TB
- New cases only
- Gender, both
- Consent to enroll in the study.

Exclusion Criteria:

- Hypercalcaemia (serum calcium > 2.6 mmol/L) identified at baseline
- Taking vitamin D
- Pregnant and lactating
- Any known liver or kidney function abnormality, malignancy

HIV testing will not be done since HIV prevalence in Bangladesh is very low and incidence of HIV among TB patients is even lower [23, 24].

Study Design: The study will be a randomized, double blind (Subject, Caregiver, Investigator, Outcomes Assessor), placebo control trial for 2 months. It will also be a safety and efficacy phase III study. The study will have 4x4 factorial design with 4-cell interventions. Enrolled patients will be randomized into the following four treatment arms in a 1:1:1:1 ratio:
Group 1: PBA
Group 2: Vitamin D3 (Cholecalciferol)
Group 3: PBA plus vitamin D3
Group 4: Placebo

Primary Outcome Measures:
- Proportion of pulmonary TB patients who are culture negative in sputum at week 4.
- Difference in improvement in clinical endpoints consisting of cough remission, chest x-ray clearance, fever remission and weight increase at 2 months.

Secondary Outcome Measures:
- Sputum smear conversion time; Time Frame: weekly up to week 12; then at week 24
- Radiological improvement [percent lung involvement on CXR at 3 months]. Time frame: week 0, 8, 12 and 24
- Cough clearance; Time frame: weekly up to week 12; then at week 24
- Weight gain; Time frame: weekly up to week 12, then at week 24
- Change in plasma PBA concentrations; Time frame: week 0, 4, 8, 12
- Change in plasma 25(OH)D_3 concentration; Time frame: week 0, 4, 8, 12, 24
- Clinical failure and default independently, and 'death or clinical failure or default': Time Frame: week 24
- Hypercalcaemia (serum calcium > 2.6 mmol/L); Time Frame: week 0, 2, 4, 8, 12
- Gastrointestinal side effects: Time Frame: weekly to week 12 then at week 24
- Immunological improvement (LL-37 in macrophages); Time Frame: week 0, 4, 8, 12
- Functional immunological improvement (killing by macrophages); Time Frame: week 0, 4, 8, 12

Treatment and Monitoring: According to the national TB program guidelines, clinical evaluation, smear microscopy for acid fast bacilli (AFB), chest x-ray (CXR) are done at 2 months from the time of anti-TB drug initiation in order to change drug regimen from 4 FDR (fixed dose regimen) to 2 FDR. Erythrocyte sedimentation rate (ESR), total and differential count (TC, DC) in blood are additionally done at 2 months. Depending on the improvement (smear negative and CXR) the drug regimen is switched from 4 FDR to 2FDR. At 2-months follow-up, if a patient is still smear positive and/or the patient is not improved clinically, and x-ray is not improved, the patient is continued on full 4 FDR treatment and advised for a follow-up visit at the 3rd month (12 weeks), 4th month (16 week), and 5th month. Smear conversion is checked at each time point. If the sputum smears are still positive during the 5th month, this constitutes treatment failure (see definition below). Additionally, the drug sensitivity

pattern (not part of National TB program and will be done for the study purpose only) is usually available by 3rd month. Based on antibiotic sensitivity pattern, drug regimen may be change if required.

Definition of clinical recovery: Absence of clinical symptoms with radiological improvement (regression/resolution of X-ray findings of at least 2/3rd of the original lesion) after assigned treatment, smear negative and weight gain at 2 months.

Definition of treatment failure: Treatment failure is defined as non-resolution of clinical and X-ray findings and sputum smear positive after 5 month of anti-TB treatment for category I patients.

Dose of interventions and Dose preparation
5,000 IU of vitamin D_3 (cholecalciferol) daily for 2 months: We have already initiated a contact with a Swedish company that can provide with droplets of 660 IU together with placebo that are produced according to GMP. The liquid formulation of vitamin D and placebo will be similar in appearance and taste. ***(Dose to be fixed after phase I trial)*** *PBA twice daily:* We plan to use sodium phenylbutyrate. Ammonaps® from Swedish Orphan AB is a registered drug containing 500 mg phenylbutyrate that is delivered in tablets. The Companies will prepare and supply doses in bottles. The PBA and placebo tablets will be similar in appearance and taste. A 3rd party will label vitamin D, PBA or placebo in bottles (A, B, C and D) and keep the information confidential and will only release the information after the completion of the trial.

The existence of gender-specific prevalence of tuberculosis in Bangladesh as well as in many other countries is well known and the female/male ratio of <1 has been observed in all types of TB case detection [25, 26]. Thus, to control and balance the influence of sex we will use "stratified block randomization method" to randomize participants into groups that result in equal sample sizes. For our study with 4 groups involving 288 participants, a randomized block procedure would be as follows: (1) a block size of 4 is chosen at two levels: male and female, (2) possible balanced combinations with 4 subjects are calculated as 24 blocks and (3) blocks will be randomly chosen to determine the assignment of all 288 participants. This procedure will result in 72 participants (36 male and 36 female) in each of the 4 treatment groups. In this procedure it is very unlikely to have imbalances in overall treatment allocations because the total sample size is >12 times higher than the block numbers and cannot generate small participant numbers within the block.

Specimens and Methods:
Sputum will be collected (Table 2) for AFB smear and mycobacterial culture. Blood will be collected (Table 3) for measurement of PBA and vitamin D_3 levels in plasma; LL-37 transcripts and peptides in macrophage; functional measures of *in vitro* macrophage mediated killing. Standard sputum smear microscopy procedure will be performed. Sputum will be cultured for MTB on Lowenstein-Jensen medium by means of standard culture techniques. Vitamin 25(OH)D_3 will be measured by autoanalyzer Cobas e114 (Roche); PBA will be measured by LC-MS (Waters). LL-37 transcripts in macrophages will be determined by real time PCR (CFX95, BioRad); LL-37 peptides will be detected by Western blot. Macrophage culture and macrophage mediated killing of MTB (H37Ra, avirulent strain) will be done by *in vitro* assays.

Table 2. Timeline for specimen and data collection

Specimens and data	wk 1*	wk 4	wk 8	wk 12	wk 24
Sputum	■	■	■	■	■
Blood	■	■	■	■	■
Chest x-ray	■		■	■	■
Cough remission	■	■	■	■	■

Fever remission					
Weight§					

Note. *1st day of the week 1 i.e. day of enrolment. §weekly weight up to wk 12.

Table 3. Detailed description of proposed investigations and data collection

Parameters		wk 1*	wk 4	wk 8	wk 12	wk 24
Sputum	AFB smear§					
	Culture, sensitivity**					
Blood	ESR, Hb, TC, DC					
	25 (OH)D_3 levels in plasma					
	PBA levels in plasma					
	Calcium levels in serum					
	LL-37 transcripts in macrophages; peptide in supernatant					
	In vitro MTB killing capacity of macrophages					
Chest x-ray	Percent lung involvement					
Anthropometry	Weight					
Clinical	Cough, fever, appetite (gastrointestinal side effects)					

Note. *1st day of the week 1 i.e. day of enrolment. **Sensitivity will be done only once at first time point. §weekly weight up to wk 12

Macrophage isolation: PBMCs (peripheral blood mononuclear cell) will be separated from heparinised blood by Ficoll-hypaque density gradient centrifugation. PBMCs will be suspended in RPMI 1640 medium containing 10% FBS, antibiotics, glutamine, Na-pyruvate and cultured in 37°C at 5% CO_2 in a cell culture plate for the adherence of monocytes. After 30 minutes, nonadherent cells will be removed and the remaining adherent cells will be cultured for 4 days; the monocyte-derived macrophages (MDMs) will be used for determination of LL-37 peptide concentration by ELISA, for quantitative PCR to determine LL-37 transcripts and for killing experiments [27].

Bacterial killing by macrophages: Macrophages would be infected with *M. tuberculosis* H37Ra at different multiplicity of infection (MOI). After 2 hours of incubation unphagocytosed TB bacilli will be washed out and infected cells will be cultured for 24 h and thereafter harvested, lysed and resuspended in 0.5% solution of saponin. The lysate will be centrifuged at 3300x g to pellet the harvested bacteria and the supernatant will be aspirated. The pellets will be washed and resuspended in 7H9 broth and 3µCi of [^{3}H] Uracil and incubated for 24 hours. The bacteria will be then fixed with 2% paraformaldehyde for 40 minute and harvested for liquid Scintillation Counting. Viability of intracellular bacteria will also be determined by CFU (Colony forming unit) assay in agar plates [28].

Quantitative Real Time PCR (qRT-PCR): RNA will be extracted from macrophages using RNA extraction kits (Qiagen Inc. Valencia, CA, USA) (Invitrogen Life Tech. Ltd), and cDNA will be synthesized using the cDNA synthesis kit (Qiagen). For quantification the relative expression of CAMP gene (LL-37) and the housekeeping gene 18S ribosomal RNA will be measured from the different RNA extractions by real-time quantitative RT-PCR, using a flurescent probe (Applied Biosystems PRISM model 7700, ABI, Foster City, CA). The expression of 18S RNA transcript numbers will be measured by PCR reactions using the 18S RNA-Housekeeping Kit (ABI). The sequences of forward and reverse primers as designed by Primer Express (ABI) for quantification of CAMP mRNA are Cathelicidin forward, 5’-GGACCCAGACACGCCAAA-3’, Cathelicidin reverse, 5’-CACACTGTCTCCTTCACTGTGA-3’ [10, 29]. The relative quantities of the gene tested per sample will be calculated by using comparative Ct method.

Western blot of LL-37 peptides: The presence of LL-37 in lyophilized protein material macrophage lysate will be determined with Western blot analysis using the polyclonal antiserum specific for LL-37. Polypeptides will be separated by discontinuous sodium dodecyl sulfate (SDS) polyacrylamide gel electrophoresis (PAGE) using 4-12% NuPAGE Ready Gels (Invitrogen, Carlsbad, CA) in Bis-Tris w/MES buffer and will be further blotted onto polyvinylidene difluoride (PVDF) membranes by electrophoretic transfer according to the instructions from the manufacturer (Invitrogen). Overnight incubation with the primary LL-37antibody in PBS with 5% fat free milk will be done. The secondary antibody will be horseradish peroxidase conjugated (Jackson Immuno Research Laboratories, Inc., West Grove, PA) and will be incubated for 1 h in PBS with 5% fat free milk. An ECL Western blot detection system (GE Healthcare, Uppsala, Sweden) will be used to visualize the results [21].

Sample Size Calculation

In rabbit studies, the mean difference in expression of LL-37 in the gut mucosal tissue between infected and treated rabbits was 6.94 with expected SD of 3.5. Considering 4 groups and expecting a 30% increase in LL-37 expression in tissues when treated with PBA + vitamin D along with standard anti-TB treatment, considering 5% level of significance and 80% power the sample size will be 62 per group. Considering a loss to follow-up of 15%, the sample size in each group will be 72. Thus total patients will be (72x4) 288 patients.

Facilities Available

Describe the availability of physical facilities at site of conduction of the study. For clinical and laboratory-based studies, indicate the provision of hospital and other types of adequate patient care and laboratory support services. Identify the laboratory facilities and major equipment that will be required for the study. For field studies, describe the field area including its size, population, and means of communications.

Patient recruitment and the clinical trial will be done in the National Institute of Diseases of the Chest and Hospital (NIDCH). The hospital has 330 beds and an average of 100 TB patients (all categories) is enrolled daily. The hospital has the technical facilities to carry out the necessary work for the proposed clinical trial including sputum smear, mycobacterium culture and sensitivity, chest x-ray, body weight and Intensive Care Unit (ICU). Pregnancy testing will be done using kits at the NIDCH hospital. In the TB Laboratory of ICDDR,B sub-samples will be assessed for microscopy and culture. Equipment necessary to measure calcium & vitamin D in plasma/serum (autoanalyzer Cobas e114 (Roche), perform macrophage cell separation and culture (Laminar flow hood, CO_2 incubator), macrophage-mediated killing of MTB (CO_2 incubator), peptide extraction, RNA and cDNA preparation, lyophilisation are available in Nutritional Biochemistry Lab and TB lab at ICDDR,B. The following measurements will be done at the Karolinska Insitutet, Sweden: facilities are available for determining concentration of PBA by LC-MS (Waters), LL-37 transcripts in macrophages by real time qPCR (PRISM) and peptides in supernatant by Western blot.

Data Safety Monitoring Plan (DSMP)

All clinical investigations (biomedical and behavioural intervention research protocols) should include the Data and Safety Monitoring Plan (DSMP) to provide the overall framework for the research protocol's data and safety monitoring. It is not necessary that the DSMP covers all possible aspects of each element. When designing an appropriate DSMP, the following should be kept in mind.

a) All investigations require monitoring;
b) The benefits of the investigation should outweigh the risks;
c) The monitoring plan should commensurate with risk; and
d) Monitoring should be with the size and complexity of the investigation.

Safety monitoring is defined as any process during clinical trials that involves the review of accumulated outcome data for groups of patients to determine if any treatment procedure practiced should be altered or not.

All relevant study data will be entered into a computer using data management software. Data will be entered twice to ensure accuracy of data. Data will also be collected from the Case Report Form and entered into the computer. Additionally, data will be recorded on standardized paper forms. Substantial effort will be made to avoid missing data and to track participants for missing follow-up visits. Raw data and data bases will be maintained under restricted access conditions to ensure confidentiality and privacy of study participants.

A Data and Safety Monitoring Board (DSMB) will be formed to monitor any adverse event and implementation of the study activity.

Data Analysis

Describe plans for data analysis. Indicate whether data will be analysed by the investigators themselves or by other professionals. Specify what statistical software packages will be used and if the study is blinded, when the code will be opened. For clinical trials, indicate if interim data analysis will be required to determine further course of the study.

Statistical analyses will be done using the statistical software SPSS 17.0 (SPSS-PASW Advanced Statistics Windows 17.0.2). The above-mentioned outcome variables will be evaluated to study the effect of vitamin D and PBA supplementation on- (a) Sputum smear conversion time; (b) radiological improvement (c) Cough clearance; (d) Weight gain; (e) plasma PBA concentrations; (f) plasma 25(OH)D_3 concentration; (g) Immunological improvement (LL-37 in macrophages and mycobacterial killing by macrophages). Comparisons between groups will be done by Student's t-test for two-group comparisons, one-way ANOVA for multi-group comparisons, and two-way ANOVA to identify possible interaction between the two independent variables. ANCOVA will be used for between-group comparisons and covariates may be used in ANCOVA to adjust for baseline levels of variables that might modify the treatment outcome (e.g., body weight, age, baseline serum vitamin D). Transformations of data will be made where needed.

Ethical Assurance for Protection of Human Rights

Describe the justifications for conducting this research in human participants. If the study needs observations on sick individuals, provide sufficient reasons for using them. Indicate how participants rights will be protected, and if there would be benefit or risk to each participants of the study.

Risk of drawing blood from patients is no greater than minimal risk. Other than momentary pain and a very small chance of bruising at the site of insertion of the needles, drawing blood will not be more than minimal risk. To minimize the chance of infection, we will take aseptic precautions and use disposable, sterile syringes and needles for drawing blood.

Safety considerations: Vitamin D: Studies in adults with daily doses of up to 250 µg (10,000 IU/day) vitamin D_3 for several months did not lead to adverse changes in serum calcium; average serum 25(OH)D concentrations were 220 nmol/L, and the dose was considered as the safe upper limit [30]. Thus, 5,000 IU of cholecalciferol (125 µg) daily is considered to be safe.
Phenylbutyrate: Our group demonstrated that PBA cleared *Shigella* infection in a rabbit model, when the animals were dosed 25.5 mg/kg, two times daily. Based on the observed induction of cathelicidin (LL-37) in the rabbit, a dose corresponding to that used for rabbits was calculated by allometric scaling for use in the current study. The calculations suggest that the effective dose for a 70 kg human would be approximately 700 mg and for a 55 kg human 600 mg of PBA. At this time there is no information on the dose needed to induce LL-37 in humans other than the rabbit data. Based on animal data, safety and simplicity we decided to use the 500 mg tablets available on the market under the brand name Ammonaps (Swedish Orphan AB). The dose for TB patients will be decided after the phase I trial in 9

healthy subjects. Three doses will be tested, 250 mg PBx2 + 5000 IU vitamin D3, 500 mg PBx2 + 5000 IU vitamin D3 and 1000 mg PBx2 + 5000 IU vitamin D3 for 4 days in 3 groups of participants. Blood will be collected for assessment of LL-37 in macrophage extracts. Adverse events if any will be monitored by the DSMB. PBA has been on the market as a therapeutic indicated as a chronic management for urea cycle disorders since 1997. It is available as granulate powder and 500 mg tablets under the brand names Ammonaps (Swedish Orphan AB) and Buphenyl (Medicis Pharmaceutical Corporation). The use of the pharmaceutical is indicated for children weighing more than 20 kg and for adults. The usual total daily dose for patients with urea cycle disorders is 450 – 600 mg/kg/day in patients weighing less than 20 kg, or 9.9 – 13.0 g/m²/day in larger patients. The tablets and powder are to be taken in equally divided amounts with each meal or feeding (i.e., three to six times per day). The dose that has been determined to be safe for use in humans with urea cycle disorders is about 20 fold higher than suggested as an adjunctive treatment for tuberculosis in the current proposal. There are, therefore, no reasons for concern regarding the safety of the treatment.

Toxicity management and side-effects: If patients are found unable to tolerate the treatment or any specific complaints are reported by the patients, symptoms will be noted and liver function test will be performed to monitor the side effects of drugs. This will be done by measuring hepatic function tests (alkaline phosphatase (ALP), aspartate transaminase (AST) alanine transaminase (ALT), gamma glutamyl transferase (γGT), bilirubin) after initiation of chemotherapy at follow-up times when complaints are reported. Accordingly, study physician will notify study investigator clinicians (SMMK, AMM) and will take prompt necessary actions.

Use of Animals

Describe if and the type and species of animals to be used in the study. Justify with reasons the use of particular animal species in the experiment and the compliance of the animal ethical guidelines for conducting the proposed procedures.

N/A

Literature Cited

Identify all cited references to published literature in the text by number in parentheses. List all cited references sequentially as they appear in the text. For unpublished references, provide complete information in the text and do not include them in the list of Literature Cited. There is no page limit for this section, however, exercise judgment in assessing the "standard" length.

1. Smith PE, Moss AR. Tuberculosis: Pathogenesis, Protection, and Control. . Epidemiology of tuberculosis. Washington, D.C.: ASM, 1994:47-60
2. WHO annual report on global TB control- summary. Wkly Epidemiol Rec. Geneva, 2003:122–128
3. Zasloff M. Antimicrobial peptides in health and disease. N Engl J Med 2002;347:1199-200
4. Nizet V, Ohtake T, Lauth X, et al. Innate antimicrobial peptide protects the skin from invasive bacterial infection. Nature 2001;414:454-7
5. Salzman NH, Ghosh D, Huttner KM, Paterson Y and Bevins CL. Protection against enteric salmonellosis in transgenic mice expressing a human intestinal defensin. Nature 2003;422:522-6
6. Ganz T. Defensins: antimicrobial peptides of innate immunity. Nat Rev Immunol 2003;3:710-20
7. Gudmundsson GH, Agerberth B. Biology and expression of the human cathelicidin LL-37. . In: Devine DA, Hancock REW, eds. Mammalian host defence peptides. . Cambridge: Cambridge University Press, 2004:139-160
8. Wang TT, Nestel FP, Bourdeau V, et al. Cutting edge: 1,25-dihydroxyvitamin D3 is a direct inducer of antimicrobial peptide gene expression. J Immunol 2004;173:2909-12
9. Weber G, Heilborn JD, Chamorro Jimenez CI, Hammarsjo A, Torma H and Stahle M. Vitamin D induces the antimicrobial protein hCAP18 in human skin. J Invest Dermatol 2005;124:1080-2
10. Liu PT, Stenger S, Li H, et al. Toll-like receptor triggering of a vitamin D-mediated human antimicrobial response. Science 2006;311:1770-3

11. Davies PD. A possible link between vitamin D deficiency and impaired host defence to Mycobacterium tuberculosis. Tubercle 1985;66:301-6
12. Frieden TR, Sterling TR, Munsiff SS, Watt CJ and Dye C. Tuberculosis. Lancet 2003;362:887-99
13. Zasloff M. Fighting infections with vitamin D. Nat Med 2006;12:388-90
14. Williams B, Williams AJ and Anderson ST. Vitamin D deficiency and insufficiency in children with tuberculosis. Pediatr Infect Dis J 2008;27:941-2
15. Rhodes SG, Terry LA, Hope J, Hewinson RG and Vordermeier HM. 1,25-dihydroxyvitamin D3 and development of tuberculosis in cattle. Clin Diagn Lab Immunol 2003;10:1129-35
16. Brodie MJ, Boobis AR, Hillyard CJ, et al. Effect of rifampicin and isoniazid on vitamin D metabolism. Clin Pharmacol Ther 1982;32:525-30
17. Nnoaham KE, Clarke A. Low serum vitamin D levels and tuberculosis: a systematic review and meta-analysis. Int J Epidemiol 2008;37:113-9
18. Islam D, Bandholtz L, Nilsson J, et al. Downregulation of bactericidal peptides in enteric infections: a novel immune escape mechanism with bacterial DNA as a potential regulator. Nat Med 2001;7:180-5
19. Bergman P, Johansson L, Asp V, et al. Neisseria gonorrhoeae downregulates expression of the human antimicrobial peptide LL-37. Cell Microbiol 2005;7:1009-17
20. Schauber J, Svanholm C, Termen S, et al. Expression of the cathelicidin LL-37 is modulated by short chain fatty acids in colonocytes: relevance of signalling pathways. Gut 2003;52:735-41
21. Raqib R, Sarker P, Bergman P, et al. Improved outcome in shigellosis associated with butyrate induction of an endogenous peptide antibiotic. Proc Natl Acad Sci U S A 2006;103:9178-83
22. Burlina AB, Ogier H, Korall H and Trefz FK. Long-term treatment with sodium phenylbutyrate in ornithine transcarbamylase-deficient patients. Mol Genet Metab 2001;72:351-5
23. Alam MS, Sarker MS, Mahmud AM, et al. Low HIV infection rates among tuberculosis patients in Dhaka, Bangladesh. Int J STD AIDS 2005;16:86-8
24. Shamputa IC, Van Deun A, Salim MA, et al. Endogenous reactivation and true treatment failure as causes of recurrent tuberculosis in a high incidence setting with a low HIV infection. Trop Med Int Health 2007;12:700-8
25. SAARC. Gender Differences among Tuberculosis Patients in National TB Control Programmes within SAARC Countries. Kathmandu, Nepal: SAARC Tuberculosis Centre, 2004:1-14
26. Zaman K, Yunus M, Arifeen SE, et al. Prevalence of sputum smear-positive tuberculosis in a rural area in Bangladesh. Epidemiol Infect 2006;134:1052-9
27. Ong PY, Ohtake T, Brandt C, et al. Endogenous antimicrobial peptides and skin infections in atopic dermatitis. N Engl J Med 2002;347:1151-60
28. Liu PT, Stenger S, Tang DH and Modlin RL. Cutting edge: vitamin D-mediated human antimicrobial activity against Mycobacterium tuberculosis is dependent on the induction of cathelicidin. J Immunol 2007;179:2060-3
29. Tsukasaki K, Tanosaki S, DeVos S, et al. Identifying progression-associated genes in adult T-cell leukemia/lymphoma by using oligonucleotide microarrays. Int J Cancer 2004;109:875-81
30. Hathcock JN, Shao A, Vieth R and Heaney R. Risk assessment for vitamin D. Am J Clin Nutr 2007;85:6-18

Dissemination and Use of Findings

Describe explicitly the plans for disseminating the accomplished results. Describe if and how the research findings would be shared with stakeholder, identifying them if known, and the mechanism to be used. Also describe what type of publication is anticipated: working papers, internal (institutional) publication, international publications, international conferences and agencies, workshops etc. Indicate, if the project is linked to the Government of the People’s Republic of Bangladesh through a training programme or a collaborative arrangement.

Research findings will be published in international journals to make the results available to all researchers in the relevant fields and will be presented at national, regional and international conferences. Additionally, important results will be disseminated through working papers and policy reports.

Collaborative Arrangements

Briefly describe if this study involves any scientific, administrative, fiscal, or programmatic arrangements with other national or international organizations or individuals. Indicate the nature and extent of collaboration and include a letter of agreement between the applicant or his/her organization and the collaborating organization.

The proposed clinical trial is a collaborative study between three institutes, namely the NIDCH (Bangladesh government), the ICDDR,B (Non-government) and the Karolinska Institutet (Swedish). The clinical trial will be conducted at the Inpatient Ward of the NIDCH under the direct supervision of Dr A. Mustaba; sputum samples will be processed for smear-microscopy, culture and sensitivity at the NIDCH Lab supervised by Dr M. Kamal. Blood samples will be sent to the Nutritional Biochemistry Lab at ICDDR,B for processing, lab investigations and storage for further shipment to KI. The overall supervision and work at ICDDR,B will be managed by Study PI (R Raqib) and the Lab investigations at KI will be supervised by Dr Birgitta Agerberth.

Itemized specific tasks for each listed investigator:

1. R Raqib: Designing and planning of study, overall supervision of work in the lab and clinic, data analysis, writing up of manuscript.
2. B. Agerberth: Designing and planning of study, coordination in obtaining PBA and vitamin D from pharmaceutical, supervision of lab work, data analysis, writing up of manuscript.
3. M. Kamal: Supervision of microbiological lab work; scientific and academic feedback.
4. A. Mustaba and M. Naimul Hoque: Supervision & management of clinical trial; clinical data analysis; scientific and academic feedback.
5. Zeaur Rahim: Scientific and academic feedback.
6. D Mondal: Scientific and academic feedback; data analysis.
7. J. Andersson: Scientific and academic feedback, especially on TB clinical trials.
8. G. Guðmundsson: Scientific and academic feedback, especially on *in vitro* assays & work related to PBA.
9. Arifuzzaman & Project Research Physician: Selection & recruitment of TB patients, routine clinical management, monitoring of patients, anti-TB therapy and adjunctive therapy.
10. Nurses: Patient care, provide doses of supplement & drug administration and checking compliance; blood collection; anthropometric measurement.
11. Research Officers: Management of day-to-day specimen collection and processing, carry out experiments specific for the protocol, data collection, data entry and compilation.
12. Research Assistants: Motivate patients for follow-up visits, conduct Lab assays for MTb culture.
13. Lab attendants: sample collection from patients, washing and cleaning of laboratory ware, autoclaving and discarding infected materials. When required, visiting patients at their respective homes to encourage patients to come to the Hospital for follow-up visits.

Sequence of tasks within time frame:

	6 m	12 mo	18 mo	24 mo
Initial set up				
Patient enrolment with follow-up				
Laboratory assays				
Data analysis and writing up				

Budget Justifications

Please provide one page statement justifying the budgeted amount for each major item, including the use of human resources, major equipment, and laboratory services.
