# Supplementary material for: Significant Effects of Oral Phenylbutyrate and Vitamin D3 Adjunctive Therapy in Pulmonary Tuberculosis: A Randomized Controlled Trial
Source: PLoS One. 2015 Sep 22;10(9):e0138340. doi: 10.1371/journal.pone.0138340 (PMC4578887; doi:10.1371/journal.pone.0138340)
Supplement: S1 Table — (DOCX) [file pone.0138340.s003.docx]

**S1 Table**. **Clinical parameters used for clinical scores**

| **Clinical features** | **Assigned scores** |
| --- | --- |
| **Self-reported symptoms** |  |
| Cough | 1 |
| Haemoptytis | 1 |
| Chest pain | 1 |
| Dyspnea/ shortness of breath | 1 |
| Anorexia | 1 |
| **Clinical assessments** |  |
| Anemia | 1 |
| Fever (≥37·8° C) | 1 |
| **Chest x-ray involvement*** | 4 |
| Lung involvement >75% | 4 |
| Lung involvement 51%-75% | 3 |
| Lung involvement 25%-50% | 2 |
| Lung involvement 01%-25% | 1 |
| Others^§^ | 0·5 |
| Total scores | 11·5 |

^§^Other symptoms include auscultatory findings, tachycardia / palpatition, general weakness and vertigo. *TB score excluding chest x-ray score was determined at week 0-4, 6, 8, 10, 12 and 24. Since chest x-ray was performed at week 0, 8, 12 and 24, a TB score including chest x-ray was determined separately for these four time points.
