# Supplementary material for: Significant Effects of Oral Phenylbutyrate and Vitamin D3 Adjunctive Therapy in Pulmonary Tuberculosis: A Randomized Controlled Trial
Source: PLoS One. 2015 Sep 22;10(9):e0138340. doi: 10.1371/journal.pone.0138340 (PMC4578887; doi:10.1371/journal.pone.0138340)
Supplement: S2 Table — (DOCX) [file pone.0138340.s004.docx]

**S2 Table. Differences between baseline characteristics when randomized patients were compared to potentially eligible patients who declined or had to be excluded from the study.**

| **Features** | **Randomized patients**  **N=251 (%)** | **Drop out from study**  **N=37 (%)** | ***P* value** |
| --- | --- | --- | --- |
| Males | 154 (53·5%) | 26 (9·0%) | 0·472 |
| History of contacts | 72 (25·0%) | 14 (4·7%) | 0·376 |
| BCG given | 171 (59·4%) | 27 (9·4%) | 0·944 |
| Age, years | 26·2±8·4 | 28·3±9·7 | 0·146 |
| Weight, kg | 44·0±8·4 | 44·4±8·4 | 0·762 |
| ESR, mm 1^st^ hr | 56·5±32·8 | 59·5±33·3 | 0·611 |
| Hb, gm/dl | 11·6±1·8 | 11·5±1·7 | 0·649 |
| WBC, 1x10^3^/cmm | 10·64±2·82 | 10·93±2·57 | 0·550 |

Data given as number with percentage in parentheses or as mean ± standard deviation. Significance p≤0.05. BCG, Bacillus Calmette–Guérin.; ESR, erythrocyte sedimentation rate; Hb, hemoglobin; WBC, white blood cells.
