# Supplementary material for: Significant Effects of Oral Phenylbutyrate and Vitamin D3 Adjunctive Therapy in Pulmonary Tuberculosis: A Randomized Controlled Trial
Source: PLoS One. 2015 Sep 22;10(9):e0138340. doi: 10.1371/journal.pone.0138340 (PMC4578887; doi:10.1371/journal.pone.0138340)
Supplement: S3 Table — (DOCX) [file pone.0138340.s005.docx]

**S3 Table. Vitamin D (25-hydroxyvitamin D_3_) status of TB patients at baseline and at 8 and 24 weeks after initiation of adjunctive therapy.**

| **Baseline** | **Placebo (n=64)** | **PBA (n=58)** | **vitD_3_ (n=62)** | **PBA+vitD_3_ (n=65)** |
| --- | --- | --- | --- | --- |
| Deficient, <30 nmol/L | 40 (62.5%) | 41 (70.7%) | 46 (74.2%) | 39 (60.0%) |
| Insufficient, 30-50 nmol/L | 19 (29.7%) | 13 (22.4%) | 8 (12.9%) | 20 (30.8%) |
| Sufficient, >50 nmol/L | 5 (7.8%) | 4 (6.9%) | 8 (12.9) | 6 (9.2%) |
| **Week 8** |  |  |  |  |
| Deficient, <30 nmol/L | 39 (60.9%) | 40 (69.0%) | 2 (3.2%) | 0 (0.0%) |
| Insufficient, 30-50 nmolLL | 16 (25.0%) | 17 (29.3%) | 2 (3.2%) | 0 (0.0%) |
| Sufficient, >50 nmol/L | 9 (14.1%) | 1 (1.7%) | 58 (93.5%) | 65 (100.0%) |
| **Week 24** | | | | |
|  | **Placebo (n=55)** | **PBA (n=52)** | **vitD_3_ (n=52)** | **PBA+vitD_3_ (n=57)** |
| Deficient, <30 nmol/L | 34 (61.8%) | 29 (55.8%) | 4 (7.7%) | 2 (3.5%) |
| Insufficient, 30-50 nmolLL | 15 (27.3%) | 15 (28.8%) | 15 (28.8%) | 17 (29.8%) |
| Sufficient, >50 nmol/L | 6 (10.9%) | 8 (15.4%) | 33 (63.5%) | 38 (66.7%) |
