# Supplementary material for: Significant Effects of Oral Phenylbutyrate and Vitamin D3 Adjunctive Therapy in Pulmonary Tuberculosis: A Randomized Controlled Trial
Source: PLoS One. 2015 Sep 22;10(9):e0138340. doi: 10.1371/journal.pone.0138340 (PMC4578887; doi:10.1371/journal.pone.0138340)
Supplement: S1 Text — (PDF) [file pone.0138340.s006.pdf]

# CONSENT FORM

Protocol Number: **PR-09068**

Protocol Title: **Clinical Trial of Oral Phenylbutyrate & Vitamin D Adjunctive Therapy in Pulmonary Tuberculosis in Bangladesh: a Pilot Study.**

Investigator's name: **Rubhana Raqib**

Organization: **ICDDR,B**

## Purpose of the research

Tuberculosis is a deadly infectious disease that is responsible for about 3 million deaths annually in the world. It is caused by *Mycobacterium tuberculosis*. Recent analysis of global burden of TB revealed that Bangladesh ranks as the 6th highest among 212 countries. Treatment with anti-TB drugs takes a long time, about 6-9 months. Our aim is to test a combination of vitamin D and phenylbutyrate together with the regular anti-TB treatment to assess whether the duration of treatment can be shortened substantially and improve immunity amongst the TB patients in Bangladesh. The study requires enrolment of about 288 TB patients. The results of this study may provide information on further improvement of TB treatment and will benefit the society in the future.

## Why are we inviting you to participate in the study?

You are being invited to participate in a research study because you have pulmonary TB disease.

## What is expected from the participants?

If you decide to volunteer for the study, you will be asked to comply with the following procedures:

1. After entering into the study, we will collect your clinical history (cough, fever, weight loss, chest x-ray etc) in the day of enrolment.
2. We will collect sputum sample from you on the day of enrolment to identify TB germs by microscopy and culture. We will also collect sputum every week thereafter to check for decrease in bacterial load. You will be given a cup for collecting sputum (deep cough).
3. We will collect 12 ml of blood each time from you in every month (4 times) to see if there is any improvement in markers of immunity and on the last follow up visit (6th month) we will collect 3 ml blood to measure vitamin D and calcium levels only. A needle will be inserted in the vein of your arm and blood will be obtained.
4. After collection of blood, you will receive oral dose of supplement (Phenylbutyrate only or Vitamin D only or Phenylbutyrate + vitamin D) or placebo (no supplement) capsules. The capsules selected for your intake will be determined in a lottery. The capsules will be unlabeled and in each capsule you may receive either placebo or supplement per day. We will not disclose the identity of the supplement during the study period.
5. Once enrolled, field workers will deliver weekly dose of either placebo or supplement capsules to you in person and will check the compliance by card and left over capsules. The daily supplementation will continue up to 2-months starting from enrolment.
6. You will receive the standard drug regimen for tuberculosis from DOTS center as usual. According to the standard practice, you will be required to visit the DOTS center to take the anti-TB drug. There will be no modification of your usual care that you receive from the centre.
7. When you come to DOTS center for your week's supply of anti-TB drugs, field workers will provide you with week's supply of our study capsules. During that time, the study physician

will ask you some questions about your physical condition (cough, fever, appetite and gastrointestinal side effects etc) to obtain data on weight and illness in the previous week. The interview process will take about 8-10 minutes.

8. The whole study will take approximately 6 months to complete.

**<u>This is the figure to show what data/specimen will be collected and when:</u>**

| Parameters | | W0 | W1 | W2 | W3 | W4 | W5 | W6 | W7 | W8 | W9 | W10 | W11 | W12 | W24 |
|---|---|---|---|---|---|---|---|---|---|---|---|---|---|---|---|
| Clinical history | X-ray | | | | | | | | | | | | | | |
| | cough, fever, Wt etc | | | | | | | | | | | | | | |
| Sputum | AFB smear | | | | | | | | | | | | | | |
| | Culture | | | | | | | | | | | | | | |
| Blood | $Ca^{2+}$, VitD & immune markers | | | | | | | | | | | | | | |

**Risks**

If you participate in this study there are no major risks associated with it. Collection of blood from a vein can cause some discomfort, bruising or momentary mild pain. During insertion of the needle, slight redness of the skin at the needle insertion site can occur and there is a rare chance of infection. We will use sterile, disposable syringes and needles and would take all precautions to prevent these problems and avoid or minimize such risks. The amount of blood that will be taken will not affect you in any way. In this study, you will consume supplement capsules daily from enrolment until 2 months. However, there are no reports linking such doses of vitamin D and Phenylbutyrate with clinical complications. No toxic or adverse effects were seen with large intakes of vitamin D from supplements in healthy people in previous studies. Phenylbutyrate is used in much higher concentration in adults and children for other diseases that have not been shown to cause any adverse effect. If you face any type of complications during the study we will take immediate actions.

**Benefits**

There will be no direct benefit to you. You will continue receiving the routine treatment from DOTS while participating in the study. This study will benefit the society in general when results are available and treatment can be improved; it will be particularly useful for people in TB endemic countries. Additionally, this study may help to find out whether vitamin D and Phenylbutyrate supplementation can improve immunity to fight against MTB.

**Privacy, anonymity and confidentiality**

The information obtained from this study will be preserved at ICDDR,B. Names, personal information, all medical records and results of the laboratory tests will be kept confidential, under lock and key. Only the study investigators of this study and any law-enforcing agency in the event of necessity would have an access to the information. We will provide you results of any or all tests performed on you, and would be happy to answer your questions about the study. However since the study is blinded, results would be available only at the end of the study.

**Future use of information**

Different types of research can be conducted in the future from the information obtained from you. The results of these investigations will be kept in scientific databases on the Internet so that many researchers around the world could study the information. However, in such cases anonymous data will be supplied to scientific databases, which will not violate the maintenance of privacy,

anonymity and confidentiality of information identifying participants in any way. You may decide to participate in the present study only and we will not use information in other future studies.

**Right not to participate and withdraw**

Participation in this study is voluntary. You may decide to participate or not in the study. You are free to change your mind later about withdrawing from the study and quit anytime even after being enrolled. You will continue receiving the standard services of the clinic even if you withdraw from the study.

**Principle of compensation**

You will be requested to provide us with your time. There will be no cost to you to participate in this study. You will receive monetary compensation for the time you spend at the clinic; specifically you will receive 500 Taka per monthly visit (total 2,000 Taka). If you are physically injured as a direct result of research procedures, reasonable necessary medical treatment will be provided.

If you agree to participate in this study, please indicate that by putting your signature or your left thumb impression at the specified space below.

Thank you for your cooperation

_______________________________________ ____________________

Signature or left thumb impression of subject Date

_______________________________________ ____________________

Signature or left thumb impression of the witness Date

_______________________________________ ____________________

Signature of the PI or his/her representative Date

Dr. Rubhana Raqib
Scientist, LSD
Phone number: 8860523-32 Ext. 2404
Mobile No:

Mr. M. A. Salam Khan
Committee coordination secretariat
Phone: 9886498 (direct)
PABX: 8860523-32 Ext. 3206
