# Supplementary material for: Significant Effects of Oral Phenylbutyrate and Vitamin D3 Adjunctive Therapy in Pulmonary Tuberculosis: A Randomized Controlled Trial
Source: PLoS One. 2015 Sep 22;10(9):e0138340. doi: 10.1371/journal.pone.0138340 (PMC4578887; doi:10.1371/journal.pone.0138340)
Supplement: S2 Text — (PDF) [file pone.0138340.s007.pdf]

Annex I

CASE REPORT FORM

Pt ID# ______________ **Week 0: Date:** ____________

Clinical history:

General appearance: ☐ mildly ill ☐ moderately ill ☐ severely ill

Infection site: ☐ pulmonary ☐ pulmonary and/or ______________________________

Signs and Symptoms: Duration of symptoms since: ____

	Yes	No		Yes	No	
1. Fever	☐	☐	5. Contact w/active case	☐	☐	9. Weight (kg) ______________
2. Cough	☐	☐	6. Loss of appetite	☐	☐	10. Others (Specify)____________
3. Chest pain	☐	☐	7. Nausea	☐	☐	__________________________
4. Haemoptysis	☐	☐	8. Anorexia	☐	☐	

**Other information:**	Yes	No		Yes	No		Yes	No
1. Previous H/O TB	☐	☐	2. BCG given	☐	☐	3. Current steroid use	☐	☐

Laboratory:

1. AFB (score, 0-3+) ☐ 0 ☐ 1+ ☐ 2+ ☐ 3+

2. L-J culture ☐ neg ☐ pos #colonies ☐ 1+ ☐ 2+ ☐ 3+ ☐ 4+ ☐ conta/lost

3. Sensitivity: Rif _____ INH _____ ETH _____ PZA ______ STR _____

4. PPD ☐ prior positive ☐ current _______ mm ☐ not done

5. Chest X-ray __________________________ % involvement __________________________

6. Blood: ESR _________ Hb _________ TC ________ Neutrophils ________ Lymphocytes ________

Week 1: Follow up date: ____________

1. a) Received anti-TB treatment ☐ regular ☐ irregular ______________________

   b) Received supplement ☐ regular ☐ irregular ______________________

2. Fever: _____________________ 5. Appetite: __________________ 7. Joint pain:______________

3. Cough: _____________________ 6. Anorexia: __________________ 8. Jaundice: ______________

4. Weight (kg): _________________

9. AFB (score,0-3+) ☐ 0 ☐ 1+ ☐ 2+ ☐ 3+ ☐ no sputum

Comments: ____

Week 2: Follow up date: ____________

1. a) Received anti-TB treatment ☐ regular ☐ irregular ________________________
   b) Received supplement ☐ regular ☐ irregular ________________________

2. Fever: ______________________ 5. Appetite: ____________________ 7. Joint pain:_________________
3. Cough: ______________________ 6. Anorexia: ____________________ 8. Jaundice: _________________
4. Weight (kg): _________________

9. AFB (score,0-3+): ☐ 0 ☐ 1+ ☐ 2+ ☐ 3+ ☐ no sputum

Comments: ____

Week 3: Follow up date: ____________

1. a) Received anti-TB treatment ☐ regular ☐ irregular ________________________
   b) Received supplement ☐ regular ☐ irregular ________________________

2. Fever: ______________________ 5. Appetite: ____________________ 7. Joint pain:_________________
3. Cough: ______________________ 6. Anorexia: ____________________ 8. Jaundice: _________________
4. Weight (kg): _________________

9. AFB (score,0-3+): ☐ 0 ☐ 1+ ☐ 2+ ☐ 3+ ☐ no sputum

Comments: ____

Week 4: Follow up date: ____________

1. a) Received anti-TB treatment ☐ regular ☐ irregular ________________________
   b) Received supplement ☐ regular ☐ irregular ________________________

2. Fever: ______________________ 5. Appetite: ____________________ 7. Joint pain:_______________
3. Cough:______________________ 6. Anorexia: ____________________ 8. Jaundice: _______________
4. Weight (kg):_________________

9. AFB (score,0-3+): ☐ 0 ☐ 1+ ☐ 2+ ☐ 3+ ☐ no sputum

10. L-J culture: ☐ neg ☐ pos (#colonies ☐1+ ☐2+ ☐ 3+ ☐ 4+ ☐ conta/lost

12. Blood: ESR__________ Hb __________ TC ________ Neutrophils ________ Lymphocytes ___________

Comments: ____

Week 6: Follow up date: ____________

1. a) Received anti-TB treatment ☐ regular ☐ irregular ________________________
   b) Received supplement ☐ regular ☐ irregular ________________________

2. Fever: ______________________ 5. Appetite: ____________________ 7. Joint pain:_________________
3. Cough: ______________________ 6. Anorexia: ____________________ 8. Jaundice: _________________
4. Weight (kg): _________________

9. AFB (score,0-3+): ☐ 0 ☐ 1+ ☐ 2+ ☐ 3+ ☐ no sputum

Comments: ____

Week 8: Follow up date: ___________

1. a) Received anti-TB treatment ☐ regular ☐ irregular ______________________
   b) Received supplement ☐ regular ☐ irregular ______________________

2. Fever: ____________________ 5. Appetite: _________________ 7. Joint pain: _____________
3. Cough: ____________________ 6. Anorexia: _________________ 8. Jaundice: _____________
4. Weight (kg): ________________

9. AFB (score,0-3+): ☐ 0 ☐ 1+ ☐ 2+ ☐ 3+ ☐ no sputum

10. L-J culture: ☐ neg ☐ pos (#colonies ☐1+ ☐2+ ☐ 3+ ☐ 4+ ☐ conta/lost

11. Chest X-ray________________________ % involvement ____________________________

12. Blood: ESR________ Hb ________ TC _______ Neutrophils _______ Lymphocytes _________

Comments: ____

Week 10: Follow up date: ___________

1. Received anti-TB treatment ☐ regular ☐ irregular ______________________

2. Fever: ____________________ 5. Appetite: _________________ 7. Joint pain: _____________
3. Cough: ____________________ 6. Anorexia: _________________ 8. Jaundice: _____________
4. Weight (kg): ________________

9. AFB (score, 0-3+): ☐ 0 ☐ 1+ ☐ 2+ ☐ 3+ ☐ no sputum

Comments: ____

Week 12: Follow up date: ___________

1. Received anti-TB treatment ☐ regular ☐ irregular ______________________

2. Fever: ____________________ 5. Appetite: _________________ 7. Joint pain: _____________
3. Cough: ____________________ 6. Anorexia: _________________ 8. Jaundice: _____________
4. Weight (kg): ________________

9. AFB (score,0-3+): ☐ 0 ☐ 1+ ☐ 2+ ☐ 3+ ☐ no sputum

10. Chest X-ray________________________ % involvement ____________________________

Comments: ____

Week 24: Follow up date: ___________

1. Received anti-TB treatment ☐ regular ☐ irregular ______________________

2. Fever: ____________________ 5. Appetite: _________________ 7. Joint pain: _____________
3. Cough: ____________________ 6. Anorexia: _________________ 8. Jaundice: _____________
4. Weight (kg): ________________

9. AFB (score,0-3+): ☐ 0 ☐ 1+ ☐ 2+ ☐ 3+ ☐ no sputum

10. Chest X-ray__________________________ % involvement ______________________________________

Comments: ____
